# Assessment of Patterns in e-Cigarette Use Among Adults in the US, 2017-2020

**DOI:** 10.1001/jamanetworkopen.2022.23266

**Published:** 2022-07-22

**Authors:** Ellen Boakye, Ngozi Osuji, John Erhabor, Olufunmilayo Obisesan, Albert D. Osei, Mohammadhassan Mirbolouk, Andrew C. Stokes, Omar Dzaye, Omar El Shahawy, Glenn A. Hirsch, Emelia J. Benjamin, Andrew P. DeFilippis, Rose Marie Robertson, Aruni Bhatnagar, Michael J. Blaha

**Affiliations:** 1 Johns Hopkins Ciccarone Center for Prevention of Cardiovascular Disease, Johns Hopkins School of Medicine, Baltimore, Maryland; 2 The American Heart Association Tobacco Regulation and Addiction Center, Dallas, Texas; 3 Department of Medicine, MedStar Union Memorial Hospital, Baltimore, Maryland; 4 Department of Internal Medicine, Yale School of Medicine, New Haven, Connecticut; 5 Department of Global Health, Boston University School of Public Health, Boston, Massachusetts; 6 Department of Population Health, New York University School of Medicine, New York; 7 Division of Cardiology, Department of Medicine, National Jewish Health, Denver, Colorado; 8 Cardiovascular Medicine, Boston University School of Medicine, Boston, Massachusetts; 9 Department of Epidemiology, Boston University School of Public Health, Boston, Massachusetts; 10 Department of Medicine, Vanderbilt University Medical Center, Nashville, Tennessee; 11 Department of Medicine, University of Louisville School of Medicine, Louisville, Kentucky

## Abstract

**Importance:**

Updated data on the patterns of e-cigarette use among adults in the US are needed.

**Objective:**

To examine recent patterns in current and daily e-cigarette use among US adults.

**Design, Setting, and Participants:**

This repeated cross-sectional study used data from the 2017, 2018, and 2020 Behavioral Risk Factor Surveillance System, a nationally representative state-based survey of noninstitutionalized US adults. A total of 994 307 adults 18 years and older who were living in states and territories that provided data on e-cigarette use in 2017 (53 states and territories), 2018 (36 states and Guam), and 2020 (42 states and Guam) were included.

**Main Outcomes and Measures:**

The weighted prevalence of current (past 30 days) and daily e-cigarette use was estimated for each year, and changes in prevalence from 2017 to 2020 were assessed, first overall and then stratified by participant characteristics, including state or territory of residence.

**Results:**

Among 994 307 adults from states with data on e-cigarette use, 429 370 individuals (weighted 51.3% female) were participants in the 2017 survey, 280 184 (weighted 52.1% female) were participants in the 2018 survey, and 284 753 (weighted 52.1% female) were participants in the 2020 survey. The weighted proportions of young adults aged 18 to 24 years were 12.6% in 2017, 11.8% in 2018, and 11.9% in 2020. Across all 3 years, 17 035 participants (weighted, 1.0%) were American Indian or Alaska Native, 22 313 (weighted, 4.6%) were Asian, 75 780 (weighted, 12.2%) were Black, 72 190 (weighted, 15.1%) were Hispanic, 4817 (weighted, 0.2%) were Native Hawaiian, 757 140 (weighted, 65.1%) were White, 20 332 (weighted, 1.3%) were multiracial, and 6245 (weighted, 0.5%) were of other races and/or ethnicities. The prevalence of current e-cigarette use was 4.4% (95% CI, 4.3%-4.5%) in 2017, which increased to 5.5% (95% CI, 5.4%-5.7%) in 2018 and decreased slightly to 5.1% (95% CI, 4.9%-5.3%) in 2020. The recent decrease, though modest, was observed mainly among young adults aged 18 to 20 years (from 18.9% [95% CI, 17.2%-20.7%] to 15.6% [95% CI, 14.1%-17.1%]; *P* = .004). However, the prevalence of daily e-cigarette use increased consistently from 1.5% (95% CI, 1.4%-1.6%) in 2017 to 2.1% (95% CI, 2.0%-2.2%) in 2018 and 2.3% (95% CI, 2.2%-2.4%) in 2020. Among young adults aged 21 to 24 years, there was a slight, albeit insignificant, increase in the prevalence of current e-cigarette use (from 13.5% [95% CI, 12.3%-14.7%] to 14.5% [95% CI, 13.2%-15.9%]; *P* = .28) but a significant increase in the prevalence of daily e-cigarette use (from 4.4% [95% CI, 3.8%-5.1%] to 6.6% [95% CI, 5.6%-7.6%]; *P* < .001) between 2018 and 2020. State-level patterns in the prevalence of current e-cigarette use were heterogeneous, with states like Massachusetts (from 5.6% [95% CI, 4.8%-6.5%] to 4.1% [95% CI, 3.1%-5.3%]; *P* = .03) and New York (from 5.4% [95% CI, 4.9%-5.9%] to 4.1% [95% CI, 3.5%-4.7%]; *P* = .001) recording significant decreases between 2018 and 2020. In contrast, Guam (from 5.9% [95% CI, 4.5%-7.9%] to 11.4% [95% CI, 8.7%-14.8%]; *P* = .002) and Utah (from 6.1% [95% CI, 5.5%-6.7%] to 7.2% [95% CI, 6.5%-8.0%]; *P* = .02) recorded significant increases in current e-cigarette use over the same period.

**Conclusions and Relevance:**

In this study, a slight decrease in the prevalence of current e-cigarette use was found between 2018 and 2020; this decrease was mainly observed among young adults aged 18 to 20 years. In contrast, daily e-cigarette use consistently increased, particularly among young adults aged 21 to 24 years. This increase in daily use suggests greater nicotine dependence among those who use e-cigarettes, warranting continued surveillance.

## Key Points

**Question:**

What are the recent patterns in current and daily e-cigarette use among US adults?

**Findings:**

In this cross-sectional study involving 994 307 adults from US states and territories that reported data on e-cigarette use in the 2017, 2018, and 2020 Behavioral Risk Factor Surveillance System, the prevalence of current e-cigarette use increased from 4.4% to 5.5% between 2017 and 2018 but decreased slightly to 5.1% in 2020; this decrease, though modest, was observed mainly among those aged 18 to 20 years. The prevalence of daily e-cigarette use increased consistently, from 1.5% in 2017 to 2.1% in 2018 and 2.3% in 2020, with the most significant increase among adults aged 21 to 24 years.

**Meaning:**

This study found a slight reduction in current e-cigarette use but consistent increases in daily e-cigarette use, suggesting greater nicotine dependence that warrants continued surveillance.

## Teaser

This cross-sectional study uses data from the 2017, 2018, and 2020 Behavioral Risk Factor Surveillance System to assess recent state-level patterns in current and daily e-cigarette use among US adults.

## Introduction

The prevalence of e-cigarette use (commonly referred to as vaping) among adults in the US has increased over the past several years.^1,2,3^ Previous studies^1,2^ examining patterns in e-cigarette use among US adults from 2016 to 2018 reported an increased prevalence of current and daily e-cigarette use, with the most substantial increase observed among young adults aged 18 to 24 years. Of public health concern, the prevalence of e-cigarette use among individuals who have never used combustible cigarettes also increased significantly over the same period.^1,2^

Until recently, e-cigarette use among youths was also increasing and surpassed the use of combustible cigarettes in 2014.^4,5,6^ More recent studies^7,8^ have reported reductions in e-cigarette use among middle school and high school students in the US. Although the reasons for this decrease remain unclear, a possible explanation may be the implementation of the Tobacco to 21 Act (Tobacco-21),^9^ which raised the minimum age for e-cigarette sales from 18 years to 21 years, potentially discouraging e-cigarette use among youths.^10^ Other explanations may be the ban on flavored cartridge–based e-cigarettes that appeal to youths^11^ and the reports of e-cigarette– or vaping product–associated lung injuries.^12^ All of these events occurred in late 2019 to early 2020 and may partly account for the observed decrease in e-cigarette use among youths. However, it is unknown whether e-cigarette use among adults, particularly young adults, has also decreased in recent years.

For the first time, through its Premarket Tobacco Product Application pathway, the US Food and Drug Administration (FDA) in October 2021 authorized the marketing of 3 e-cigarette products sold under the brand name Vuse (R. J. Reynolds Tobacco Company).^13^ The rationale for the authorization was that the potential benefit of using e-cigarette products as tobacco cessation aids for adult smokers appeared to outweigh the risk of youths using these products.^13^ However, although e-cigarettes have been reported to aid cessation among adults who smoke combustible cigarettes, their use among youths, young adults, and individuals who have never used combustible cigarettes remains of substantial public health concern.

Delineating the patterns of e-cigarette use is important to informing effective regulatory policies. Changes in e-cigarette use, particularly among young adults aged 18 to 20 years, may be used to evaluate policies such as the Tobacco-21 legislation. In addition, state-level patterns can be used to assess state-level policies. To monitor recent patterns, we used 2017, 2018, and 2020 data from one of the most extensive health surveys, the Behavioral Risk Factor Surveillance System (BRFSS), to examine recent changes in current and daily e-cigarette use among US adults.

## Methods

### Data Source, Study Sample, and Study Design

This repeated cross-sectional study used data from the 2017, 2018, and 2020 BRFSS, a nationally representative survey of noninstitutionalized adults 18 years and older in the US. Verbal informed consent was obtained from all participants in the initial BRFSS surveys. The present study was deemed exempt from review by an institutional review board because it used deidentified publicly available BRFSS data. This study followed the Strengthening the Reporting of Observational Studies in Epidemiology (STROBE) reporting guideline for cross-sectional studies.

The BRFSS uses iterative proportional fitting as a weighting method, which incorporates demographic characteristics to adjust for noncoverage and nonresponse, thereby making the data representative.^14^ In this study, we used data from all states, including the District of Columbia, and all territories (hereinafter referred to as states) that provided data on e-cigarette use in 2017 (53 states and territories), 2018 (36 states and Guam), or 2020 (42 states and Guam) (eTable 1 in the Supplement). In 2017, inclusion of the survey module containing questions about e-cigarette use was required and used by all states. However, in 2018 and 2020, this module was optional, giving states the flexibility to include the module in their state surveys based on their priorities. Therefore, not all states provided data on e-cigarette use in 2018 and 2020. No data on e-cigarette use were collected in 2019 surveys. The median survey response rate for all states was 45.1% (range, 30.6%-64.1%) in 2017, 49.4% (range, 37.3%-73.1%) in 2018, and 47.9% (range, 34.5%-67.2%) in 2020.^15,16,17^ A description of how we assessed e-cigarette and combustible cigarette use and other study measures has been published previously^18^ and is also provided in eMethods in the Supplement.

### Statistical Analysis

First, using data from all states with information on e-cigarette use in 2017, 2018, or 2020, irrespective of whether a state provided e-cigarette data for all 3 years, we calculated the weighted prevalence of current (past 30 days) and daily e-cigarette use for each year, both overall and stratified by age. In the sensitivity analysis, we used data from the 33 states that had data on e-cigarette use for all 3 years to calculate the changing prevalence of current and daily e-cigarette use. We also estimated the prevalence of current e-cigarette use for each state and examined patterns between 2017 and 2020.

For comparison, we examined patterns in combustible cigarette smoking from 2017 to 2020, first using only the states with data on e-cigarette use, then incorporating all states with data on combustible cigarette use as a sensitivity analysis. Using the 2010 US population as the standard, we obtained estimates for the following age groups: 18 to 24 years, 25 to 29 years, 30 to 34 years, 35 to 39 years, 40 to 44 years, 45 to 49 years, 50 to 54 years, 55 to 59 years, and 60 years and older. We used those estimates to calculate the age-standardized prevalence of current e-cigarette use by participant characteristics and examined patterns from 2017 to 2020.^19^

Among current smokers of combustible cigarettes, we estimated the proportion who reported attempting to quit smoking in the past year and the prevalence of current e-cigarette use among those attempting to quit in the past year. We also assessed the prevalence of sole e-cigarette use, dual e-cigarette and combustible cigarette use, and sole combustible cigarette use.

All analyses were conducted using Stata software, version 16 (StataCorp LLC). The survey command svy was used to account for the complex weighting method used by the BRFSS, and statistical significance was set at 2-sided *P* < .05.

## Results

### Patterns in e-Cigarette and Combustible Cigarette Use

A total of 994 307 adults from states with data on e-cigarette use were included. Of those, 429 370 individuals (51.3% female; 48.7% male) were participants in the 2017 survey, 280 184 individuals (52.1% female; 47.9% male) were participants in the 2018 survey, and 284 753 individuals (52.1% female; 47.9% male) were participants in the 2020 survey. The weighted proportions of young adults aged 18 to 24 years were 12.6% in 2017, 11.8% in 2018, and 11.9% in 2020. Across all 3 years, 17 035 participants (weighted, 1.0%) were American Indian or Alaska Native, 22 313 (weighted, 4.6%) were Asian, 75 780 (weighted, 12.2%) were Black, 72 190 (weighted, 15.1%) were Hispanic, 4817 (weighted, 0.2%) were Native Hawaiian, 757 140 (weighted, 65.1%) were White, 20 332 (weighted, 1.3%) were multiracial, and 6245 (weighted, 0.5%) were of other races and/or ethnicities.

**Figure 1.**
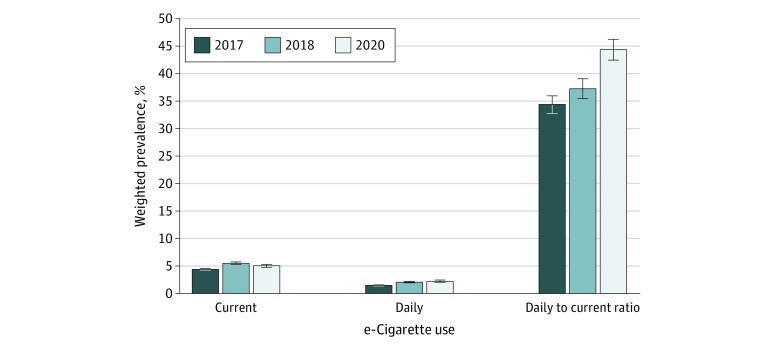
Weighted Prevalence of Current and Daily e-Cigarette Use Among US Adults in the Behavioral Risk Factor Surveillance System Whiskers represent 95% CIs.

The weighted prevalence of current e-cigarette use was 4.4% (95% CI, 4.3%-4.5%) in 2017, which increased to 5.5% (95% CI, 5.4%-5.7%) in 2018, then decreased slightly to 5.1% (95% CI, 4.9%-5.3%) in 2020. The prevalence of daily e-cigarette use increased steadily from 1.5% (95% CI, 1.4%-1.6%) in 2017 to 2.1% (95% CI, 2.0%-2.2%) in 2018 and 2.3% (95% CI, 2.2%-2.4%) in 2020. The proportion of current e-cigarette users who reported daily use therefore increased from 34.5% (95% CI, 33.1%-36.0%) in 2017 to 37.3% (95% CI, 35.5%-39.1%) in 2018 and 44.4% (95% CI, 42.5%-46.3%) in 2020 (Figure 1).

**Figure 2.**
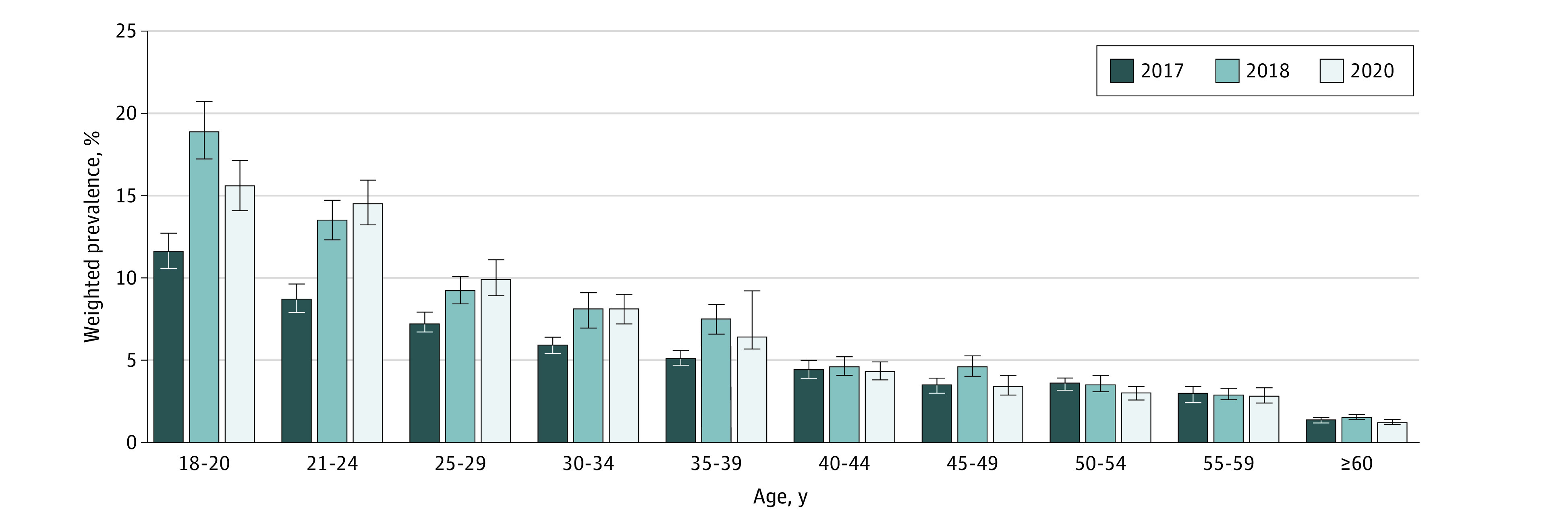
Weighted Prevalence of Current e-Cigarette Use by Age Among US Adults in the Behavioral Risk Factor Surveillance System Whiskers represent 95% CIs.

**Figure 3.**
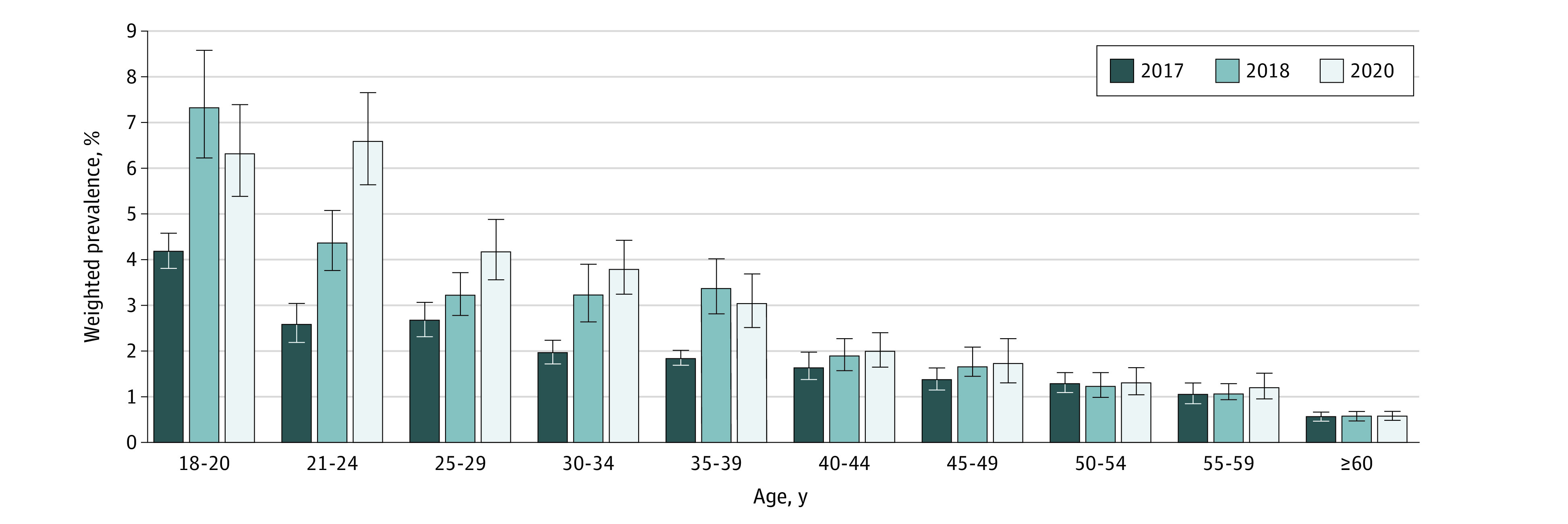
Weighted Prevalence of Daily e-Cigarette Use by Age Among US Adults in the Behavioral Risk Factor Surveillance System Whiskers represent 95% CIs.

The modest reduction in the prevalence of current e-cigarette use between 2018 and 2020 was mainly observed among young adults aged 18 to 20 years (from 18.9% [95% CI, 17.2%-20.7%] to 15.6% [95% CI, 14.1%-17.1%]; *P* = .004) (Figure 2). Among young adults aged 21 to 24 years, there was a slight, albeit insignificant, increase in the prevalence of current e-cigarette use (from 13.5% [95% CI, 12.3%-14.7%] to 14.5% [95% CI, 13.2%-15.9%]; *P* = .28) but a significant increase in the prevalence of daily e-cigarette use (from 4.4% [95% CI, 3.8%-5.1%] to 6.6% [95% CI, 5.6%-7.6%]; *P* < .001) between 2018 and 2020 (Figure 3). Results from a sensitivity analysis using data from only the 33 states with information on e-cigarette use for all 3 years revealed similar patterns in the prevalence of current and daily e-cigarette use, both overall (current use: 4.5% [95% CI, 4.4%-4.7%] in 2017, 5.5% [95% CI, 5.3%-5.7%] in 2018, and 5.2% [95% CI, 4.9%-5.4%] in 2020; daily use: 1.5% [95% CI, 1.4-1.6] in 2017, 2.1% [95% CI, 1.9%-2.2%] in 2018, and 2.3% [95% CI, 2.2%-2.5%] in 2020) and stratified by age (eg, current use for ages 18-20 years: 11.9% [95% CI, 10.6%-13.4%] in 2017, 18.6% [95% CI, 16.8%-20.5%] in 2018, and 15.5% [95% CI, 13.8%-17.3%] in 2020; daily use for ages 18-20 years: 3.5% [95% CI, 2.8%-4.3%] in 2017, 7.1% [95% CI, 6.0%-8.5%] in 2018, and 6.0% [95% CI, 5.0%-7.2%] in 2020) (eTable 2 in the Supplement).

Among the states with data on e-cigarette use, the prevalence of current combustible cigarette smoking was 16.3% (95% CI, 16.1%-16.6%) in 2017, which remained relatively stable at 16.2% (95% CI, 15.9%-16.5%) in 2018 but decreased to 14.8% (95% CI, 14.5%-15.1%) in 2020. An analysis of data from all states with information on combustible cigarette use, irrespective of whether a state had data on e-cigarette use, revealed similar decreasing patterns (16.3% [95% CI, 16.1%–16.5%] in 2017, 15.5% [95% CI, 15.3%-15.7%] in 2018, and 14.2% [95% CI, 14.0%-14.5%] in 2020). Similar to the reductions in e-cigarette use, the decrease in combustible cigarette use between 2018 and 2020 was predominantly observed among the younger age groups (eg, ages 18-20 years: from 8.7% [95% CI, 7.5%-10.1%] to 5.3% [95% CI, 4.5%-6.2%]; ages 21-24 years: from 14.4% [95% CI, 13.2%-15.5%] to 10.1% [95% CI, 9.2%-11.2%]) (eFigure 1 in the Supplement).

### Patterns in e-Cigarette Use by Participant Characteristics

The age-standardized prevalence of current e-cigarette use in 2017, 2018, and 2020 by participant characteristics is shown in Table 1. Across all years, the prevalence of current e-cigarette use was higher among men (5.5% [95% CI, 5.3%-5.7%] in 2017, 7.4% [95% CI, 7.1%-7.8%] in 2018, and 6.6% [95% CI, 6.2%-6.9%] in 2020), transgender individuals (8.6% [95% CI, 5.6%-12.9%] in 2017, 9.2% [95% CI, 6.3%-13.1%] in 2018, and 10.9% [95% CI, 7.2%-16.3%] in 2020), individuals who reported using smokeless tobacco products (8.9% [95% CI, 8.0%-9.9%] in 2017, 10.2% [95% CI, 9.1%-11.4%] in 2018, and 12.8% [95% CI, 11.5%-14.3%] in 2020) or cannabis in the past month (8.9% [95% CI, 7.4%-10.5%] in 2017, 17.4% [95% CI, 15.6%-19.4%] in 2018, and 15.7% [95% CI, 14.6%-16.9%] in 2020), and individuals who reported drinking heavily (7.9% [95% CI, 7.2%-8.6%] in 2017, 11.7% [95% CI, 10.8%-12.7%] in 2018, and 11.5% [95% CI, 10.5%-12.6%] in 2020) or binge drinking (6.9% [95% CI, 6.5%-7.3%] in 2017, 10.1% [95% CI, 9.5%-10.8%] in 2018, and 10.4% [95% CI, 9.8%-11.1%] in 2020).

**Table 1.** Age-Standardized Prevalence and Change in Absolute Prevalence of Current e-Cigarette Use Among US Adults in the Behavioral Risk Factor Surveillance System

Characteristics	Age-standardized prevalence, % (95% CI)^a^			Difference in absolute prevalence, % (95% CI)		
	2017	2018	2020	2018 vs 2017	2020 vs 2018	2020 vs 2017
Total unweighted participants, No.	429 370	280 184	284 753	NA	NA	NA
Sex						
Female	3.6 (3.4 to 3.8)	4.7 (4.4 to 4.9)	4.7 (4.4 to 5.0)	1.1 (0.7 to 1.4)	0 (−0.4 to 0.4)	1.1 (0.8 to 1.4)
Male	5.5 (5.3 to 5.7)	7.4 (7.1 to 7.8)	6.6 (6.2 to 6.9)	2.0 (1.5 to 2.4)	−0.9 (−1.3 to −0.4)	1.1 (0.7 to 1.5)
Race and ethnicity						
American Indian or Alaska Native	6.9 (5.7 to 8.4)	8.4 (6.0 to 11.2)	6.3 (4.9 to 8.2)	1.4 (−1.5 to 4.3)	−1.9 (−5.0 to 1.1)	−0.6 (−2.7 to 1.6)
Asian	2.4 (1.9 to 2.9)	3.6 (2.9 to 4.4)	3.5 (2.7 to 4.4)	1.2 (0.3 to 2.1)	−0.1 (−1.2 to 1.0)	1.1 (0.1 to 2.1)
Black	3.2 (2.8 to 3.6)	3.7 (3.3 to 4.2)	3.7 (3.1 to 4.4)	0.6 (−0.1 to 1.0)	0 (−0.8 to 0.8)	0.5 (−0.2 to 1.3)
Hispanic	2.5 (2.2 to 2.8)	4.3 (3.7 to 4.9)	3.5 (3.0 to 4.0)	1.8 (1.1 to 2.5)	−0.8 (−1.6 to 0)	1.0 (0.5 to 1.6)
Native Hawaiian	4.5 (2.5 to 7.8)	7.2 (5.5 to 9.3)	6.8 (5.1 to 9.1)	2.7 (−0.4 to 5.9)	−0.4 (−3.1 to 2.3)	2.4 (−0.9 to 5.6)
White	5.7 (5.5 to 5.9)	7.0 (6.8 to 7.3)	7.0 (6.7 to 7.3)	1.4 (1.0 to 1.7)	0 (−0.4 to 0.4)	1.3 (1.0 to 1.7)
Multiracial	7.3 (6.3 to 8.5)	9.7 (8.4 to 11.1)	7.5 (6.3 to 8.9)	2.3 (0.6 to 4.1)	−2.2 (−4.1 to −0.3)	0.2 (−1.5 to 1.9)
Sexual orientation^b^						
Bisexual	7.8 (6.2 to 9.7)	8.4 (7.1 to 9.7)	9.9 (7.8 to 12.4)	0.6 (−1.6 to 2.0)	1.5 (−1.1 to 4.2)	2.1 (−0.8 to 5.0)
Heterosexual	4.4 (4.2 to 4.6)	5.8 (5.5 to 6.1)	5.2 (4.9 to 5.4)	1.4 (1.0 to 1.8)	−0.6 (−1.0 to −0.2)	0.8 (0.4 to 1.1)
Lesbian or gay	6.6 (5.1 to 8.4)	9.0 (7.1 to 11.2)	8.3 (6.5 to 10.5)	2.4 (−0.2 to 5.0)	−0.7 (−3.5 to 2.2)	1.7 (−0.8 to 4.3)
Transgender^b^						
No	4.4 (4.2 to 4.6)	5.9 (5.6 to 6.2)	5.4 (5.2 to 5.7)	1.4 (1.1 to 1.8)	−0.5 (−0.9 to −0.1)	1.0 (0.6 to 1.3)
Yes	8.6 (5.6 to 12.9)	9.2 (6.3 to 13.1)	10.9 (7.2 to 16.3)	0.6 (−4.3 to 5.6)	1.7 (−3.9 to 7.3)	2.4 (−3.4 to 8.1)
BMI						
<18.5	7.3 (6.0 to 9.0)	7.8 (6.3 to 9.6)	8.5 (6.8 to 10.6)	0.5 (−1.7 to 2.7)	0.7 (−1.8 to 3.2)	1.2 (−1.2 to 3.6)
18.5 to <25.0	4.6 (4.4 to 4.9)	6.4 (6.1 to 6.8)	6.1 (5.7 to 6.5)	1.8 (1.4 to 2.2)	−0.3 (−0.9 to 0.2)	1.5 (1.0 to 1.9)
25.0 to <30.0	4.7 (4.4 to 4.9)	6.1 (5.7 to 6.5)	5.7 (5.3 to 6.1)	1.4 (0.9 to 1.9)	−0.4 (−1.0 to 0.2)	1.0 (0.5 to 1.5)
≥30.0	5.1 (4.7 to 5.4)	5.9 (5.5 to 6.4)	5.7 (5.3 to 6.2)	0.9 (0.3 to 1.4)	−0.2 (−0.8 to 0.4)	0.7 (0.1 to 1.2)
Marital status						
Married	3.7 (3.4 to 4.0)	4.1 (3.8 to 4.5)	4.6 (4.0 to 5.2)	0.5 (0 to 1.0)	0.5 (−0.2 to 1.2)	1.0 (0.3 to 1.0)
Divorced	6.3 (5.6 to 7.0)	10.0 (8.4 to 12.0)	7.2 (6.2 to 8.4)	3.8 (1.8 to 5.7)	−2.8 (−4.9 to −0.7)	0.9 (−0.3 to 2.2)
Widowed	9.7 (6.6 to 14.2)	7.3 (4.7 to 11.2)	7.2 (4.7 to 11.0)	−2.4 (−7.3 to 2.5)	−0.1 (−4.5 to 4.0)	−2.5 (−7.4 to 2.4)
Single	4.8 (4.5 to 5.1)	6.5 (6.1 to 6.8)	6.0 (5.5 to 6.4)	1.7 (1.2 to 2.2)	−0.5 (−1.1 to 0.1)	1.2 (0.7 to 1.7)
Member of an unmarried couple	6.2 (5.5 to 7.0)	8.7 (7.7 to 9.9)	8.5 (7.3 to 9.9)	2.5 (1.2 to 3.8)	−0.2 (−1.9 to 1.5)	2.3 (0.8 to 3.8)
Educational level						
Less than high school	5.0 (4.6 to 5.5)	6.7 (6.0 to 7.5)	6.0 (5.3 to 6.9)	1.7 (0.8 to 2.0)	−0.7 (−1.8 to 0.5)	1.0 (0.1 to 2.0)
High school or some college	5.5 (5.3 to 5.7)	7.0 (6.7 to 7.3)	6.4 (6.2 to 6.7)	1.5 (1.1 to 1.8)	−0.6 (−1.0 to −0.2)	0.9 (0.6 to 1.3)
College	2.1 (2.0 to 2.3)	3.4 (3.1 to 3.7)	3.6 (3.3 to 3.9)	1.3 (1.0 to 1.6)	0.2 (−0.2 to 0.6)	1.4 (1.1 to 1.8)
Employment status						
Employed	4.5 (4.4 to 4.7)	6.2 (5.9 to 6.4)	6.1 (5.8 to 6.4)	1.6 (1.3 to 1.9)	−0.1 (−0.5 to 0.3)	1.5 (1.2 to 1.9)
Unemployed	5.8 (5.5 to 6.2)	6.5 (6.0 to 7.1)	6.3 (5.8 to 6.8)	0.7 (0 to 1.3)	−0.3 (−1.0 to 0.5)	0.4 (−0.2 to 1.1)
Student	4.6 (3.6 to 5.9)	7.3 (5.4 to 9.7)	7.6 (4.9 to 11.6)	2.7 (0.3 to 5.1)	0.3 (−3.6 to 4.2)	3.0 (−0.4 to 6.5)
Retired	4.5 (2.5 to 7.8)	8.0 (4.6 to 13.7)	6.7 (4.0 to 10.9)	3.6 (−1.5 to 8.6)	−1.4 (−6.9 to 4.2)	2.2 (−2.1 to 6.4)
Income, poverty line, %						
<100	4.9 (4.5 to 5.4)	6.4 (5.9 to 7.0)	6.3 (5.7 to 7.0)	1.5 (0.8 to 2.2)	−0.1 (−0.9 to 0.8)	1.4 (0.7 to 2.2)
100-200	5.0 (4.7 to 5.3)	6.9 (6.4 to 7.4)	6.6 (6.1 to 7.1)	1.9 (1.3 to 2.5)	−0.3 (−1.0 to 0.4)	1.6 (1.0 to 2.2)
>200	4.2 (4.0 to 4.5)	5.8 (5.6 to 6.1)	5.2 (5.0 to 5.5)	1.6 (1.3 to 1.9)	−0.6 (−1.0 to −0.2)	1.0 (0.7 to 1.3)
Rural or urban area of residence						
Rural	NA	6.0 (5.3 to 6.9)	6.6 (5.8 to 7.6)	NA	0.6 (−0.6 to 1.8)	NA
Urban	NA	6.1 (5.9 to 6.3)	5.6 (5.4 to 5.8)	NA	−0.5 (−0.8 to −0.2)	NA
Combustible cigarette smoking						
Never	1.3 (1.2 to 1.4)	2.2 (2.1 to 2.4)	2.3 (2.1 to 2.5)	0.9 (0.7 to 1.1)	0.1 (−0.1 to 0.3)	1.0 (0.8 to 1.2)
Former	8.9 (8.3 to 9.5)	12.3 (11.4 to 13.2)	13.3 (12.3 to 14.2)	3.4 (2.4 to 4.5)	1.0 (−0.3 to 2.3)	4.4 (3.3 to 5.5)
Current	13.7 (13.1 to 14.3)	15.5 (14.7 to 16.3)	13.8 (13.0 to 14.6)	1.8 (0.8 to 2.7)	−1.7 (−2.8 to −0.6)	0.1 (−1.0 to 1.1)
Smokeless tobacco						
No	4.4 (4.2 to 4.5)	5.9 (5.7 to 6.1)	5.38 (5.2 to 5.6)	1.5 (1.2 to 1.8)	−0.5 (−0.8 to −0.2)	1.0 (0.8 to 1.3)
Yes	8.9 (8.0 to 9.9)	10.2 (9.1 to 11.4)	12.8 (11.5 to 14.3)	1.3 (−0.2 to 2.8)	2.6 (0.8 to 4.5)	3.9 (2.2 to 5.6)
Cannabis use in past mo^b^						
No	2.8 (2.6 to 3.1)	4.4 (4.0 to 4.8)	4.1 (3.9 to 4.3)	1.5 (1.1 to 2.0)	−0.3 (−0.7 to 0.2)	1.3 (0.9 to 1.6)
Yes	8.9 (7.4 to 10.5)	17.4 (15.6 to 19.4)	15.7 (14.6 to 16.9)	8.6 (6.1 to 11.0)	−1.7 (−4.0 to 0.5)	6.8 (4.9 to 8.8)
Heavy alcohol use						
No	4.3 (4.2 to 4.5)	5.6 (5.4 to 5.8)	5.2 (4.9 to 5.4)	1.3 (1.0 to 1.5)	−0.4 (−0.7 to −0.1)	0.84 (0.6 to 1.1)
Yes	7.9 (7.2 to 8.6)	11.7 (10.8 to 12.7)	11.5 (10.5 to 12.6)	3.9 (2.7 to 5.0)	−0.2 (−1.6 to 1.2)	3.7 (2.4 to 4.9)
Binge drinking						
No	3.9 (3.8 to 4.1)	4.9 (4.7 to 5.1)	4.4 (4.2 to 4.7)	1.0 (0.7 to 1.2)	−0.5 (−0.8 to −0.2)	0.5 (0.2 to 0.8)
Yes	6.9 (6.5 to 7.3)	10.1 (9.5 to 10.8)	10.4 (9.8 to 11.1)	3.3 (2.6 to 4.0)	0.3 (−0.7 to 1.2)	3.5 (2.8 to 4.3)
CVD^c^						
No	4.4 (4.3 to 4.6)	5.9 (5.7 to 6.2)	5.5 (5.3 to 5.7)	1.5 (1.3 to 1.8)	−0.4 (−0.7 to −0.1)	1.1 (0.9 to 1.4)
Yes	7.2 (6.0 to 8.5)	9.1 (7.3 to 11.3)	8.3 (6.7 to 10.3)	1.9 (−0.4 to 4.3)	−0.8 (−3.5 to 1.9)	1.2 (−1.0 to 3.3)
Asthma						
No	4.3 (4.2 to 4.5)	5.8 (5.5 to 6.0)	5.4 (5.1 to 5.6)	1.5 (1.2 to 1.7)	−0.4 (−0.7 to −0.1)	1.0 (0.8 to 1.3)
Yes	6.1 (5.7 to 6.5)	7.6 (7.0 to 8.2)	7.07 (6.5 to 7.7)	1.5 (0.8 to 2.2)	−0.5 (−1.3 to 0.3)	1.0 (0.3 to 1.7)
COPD						
No	4.3 (4.1 to 4.4)	5.7 (5.5 to 6.0)	5.4 (5.2 to 5.7)	1.5 (1.2 to 1.7)	−0.3 (−0.6 to 0)	1.2 (0.9 to 1.4)
Yes	9.4 (8.4 to 10.4)	12.2 (10.7 to 13.9)	10.6 (8.9 to 12.5)	2.9 (1.0 to 4.7)	−1.6 (−4.0 to 0.8)	1.2 (−0.8 to 3.3)
Depression						
No	3.7 (3.5 to 3.8)	5.1 (4.9 to 5.3)	4.5 (4.2 to 4.7)	1.5 (1.2 to 1.7)	−0.7 (−1.0 to −0.3)	0.8 (0.5 to 1.1)
Yes	8.3 (7.9 to 8.7)	9.8 (9.3 to 10.4)	9.9 (9.3 to 10.5)	1.5 (0.9 to 2.2)	0.1 (−0.7 to 0.9)	1.6 (0.9 to 2.4)
Cancer^d^						
No	4.5 (4.4 to 4.7)	6.0 (5.8 to 6.2)	5.5 (5.3 to 5.7)	1.5 (1.2 to 1.8)	−0.5 (−0.8 to −0.2)	1.0 (0.8 to 1.3)
Yes	7.0 (5.9 to 8.4)	8.9 (6.8 to 11.5)	10.4 (8.0 to 13.5)	1.8 (−0.8 to 4.5)	1.6 (−2.0 to 5.2)	3.4 (0.4 to 6.4)
Pregnant						
No	4.6 (4.3 to 4.9)	6.5 (6.1 to 7.0)	6.9 (6.4 to 7.4)	2.0 (1.4 to 2.5)	0.3 (−0.3 to 1.0)	2.3 (1.7 to 2.8)
Yes	3.5 (1.3 to 9.3)	3.3 (2.1 to 5.2)	5.0 (2.5 to 9.7)	−0.2 (−4.0 to 3.6)	1.7 (−2.0 to 5.4)	1.5 (−3.4 to 6.4)

Abbreviations: BMI, body mass index (calculated as weight in kilograms divided by height in meters squared); COPD, chronic obstructive pulmonary disease; CVD, cardiovascular disease; NA, not applicable.

^a^ All estimates were standardized to the 2010 adult US population.

^b^ Data were not available for the entire Behavioral Risk Factor Surveillance System data years. Reported prevalence estimates were calculated from analyses using data from states that included these questions in their questionnaires.

^c^ Composite of myocardial infarction, coronary heart disease, and/or stroke.

^d^ Skin cancer was excluded.

Across most sociodemographic subgroups, the age-standardized prevalence of current e-cigarette use decreased between 2018 and 2020, with significant reductions among men (from 7.4% [95% CI, 7.1%-7.8%] to 6.6% [95% CI, 6.2%-6.9%]; *P* < .001), multiracial individuals (from 9.7% [95% CI, 8.4%-11.1%] to 7.5% [95% CI, 6.3%-8.9%]; *P* = .03), heterosexual individuals (from 5.8% [95% CI, 5.5%-6.1%] to 5.2% [95% CI, 4.9%-5.4%]; *P* = .002), cisgender individuals (from 5.9% [95% CI, 5.6%-6.2%] to 5.4% [95% CI, 5.2%-5.7%]; *P* = .01), and individuals with income greater than 200% of the federal poverty line (from 5.8% [95% CI, 5.6%-6.1%] to 5.2% [95% CI, 5.0%-5.5%]; *P* = .002). A decrease in the prevalence of e-cigarette use between 2018 and 2020 was observed among those living in urban areas (from 6.1% [95% CI, 5.9%-6.3%] to 5.6% [95% CI, 5.4%-5.8%]; *P* = .002) but not in rural areas (from 6.0% [95% CI, 5.3%-6.9%] to 6.6% [95% CI, 5.8%-7.6%]; *P* = .32).

The prevalence of current e-cigarette use between 2018 and 2020 decreased among individuals who currently used combustible cigarettes (from 15.5% [95% CI, 14.7%-16.3%] to 13.8% [95% CI, 13.0%-14.6%]) but not among individuals who never used combustible cigarettes (from 2.2% [95% CI, 2.1%-2.4%] to 2.3% [95% CI, 2.1%-2.5%]) or previously used combustible cigarettes (from 12.3% [95% CI, 11.4%-13.2%] to 13.3% [95% CI, 12.3%-14.2%]) (Table 1). Age-stratified patterns in the prevalence of current e-cigarette use among smokers who never used combustible cigarettes are shown in eTable 3 in the Supplement.

### Patterns in e-Cigarette Use Among Smokers of Combustible Cigarettes With Quit Attempts

Among current smokers of combustible cigarettes, 58.9% (95% CI, 58.1%-59.7%) in 2017, 57.1% (95% CI, 56.1%-58.1%) in 2018, and 54.8% (95% CI, 53.7%-55.9%) in 2020 reported making quit attempts in the past year. The prevalence of current e-cigarette use among those who reported making quit attempts in the past year was 13.1% (95% CI, 12.2%-14.2%) in 2020, which represented a decrease from 17.1% (95% CI, 16.1%-18.1%) in 2018 and 16.0% (95% CI, 15.2%-16.8%) in 2017.

### Patterns in e-Cigarette and Combustible Cigarette Use

Overall, in 2020, the prevalence of sole e-cigarette use was 3.4% (95% CI, 3.2%-3.6%), the prevalence of dual e-cigarette and combustible cigarette use was 1.7% (95% CI, 1.6%-1.8%), and the prevalence of sole combustible cigarette use was 13.2% (95% CI, 12.9%-13.5%), with considerable differences by age. Although sole e-cigarette use and dual e-cigarette and combustible cigarette use were widespread among younger age groups (eg, ages 18-20 years: 12.5% [95% CI, 11.2%-13.9%] for sole e-cigarette use and 3.0% [95% CI, 2.3%-3.8%] for dual e-cigarette and combustible cigarette use; ages 21-24 years: 10.7% [95% CI, 9.6%-11.9%] for sole e-cigarette use and 3.5% [95% CI, 2.9%-4.1%] for dual e-cigarette and combustible cigarette use), sole combustible cigarette use was higher among older age groups (eg, 16.2% [95% CI, 15.2%-17.3%] for ages 50-54 years, 17.1% [95% CI, 16.0%-18.2%] for ages 55-59 years, and 11.0% [95% CI, 10.7%-11.5%] for ages ≥60 years) (eFigure 2 in the Supplement). The overall prevalence of dual e-cigarette and combustible cigarette use was 2.2% (95% CI, 2.1%-2.3%) in 2017, increasing to 2.4% (95% CI, 2.3%-2.5%) in 2018 but decreasing to 1.7% (95% CI, 1.6%-1.8%) in 2020 (eFigure 3 in the Supplement).

### Prevalence of e-Cigarette Use by State

Patterns in the prevalence of current e-cigarette use from 2017 to 2020 by state are shown in Table 2 and eFigures 4 to 6 in the Supplement. Between 2017 and 2018, most states recorded significant increases in the prevalence of current e-cigarette use. Between 2018 and 2020, Connecticut (from 5.6% [95% CI, 4.9%-6.4%] to 4.5% [95% CI, 3.8%-5.2%]; *P* = .04), Massachusetts (from 5.6% [95% CI, 4.8%-6.5%] to 4.1% [95% CI, 3.1%-5.3%]; *P* = .03), New York (from 5.4% [95% CI, 4.9%-5.9%] to 4.1% [95% CI, 3.5%-4.7%]; *P* = .001), and North Dakota (from 6.4% [95% CI, 5.3%-7.6%] to 4.5% [95% CI, 3.6%-5.6%]; *P* = .02) recorded significant decreases in the prevalence of e-cigarette use. In contrast, Guam (from 5.9% [95% CI, 4.5%-7.9%] to 11.4% [95% CI, 8.7%-14.8%]; *P* = .002) and Utah (from 6.1% [95% CI, 5.5%-6.7%] to 7.2% [95% CI, 6.5%-8.0%]; *P* = .02) recorded significant increases in the prevalence of current e-cigarette use over the same period. Kansas (from 5.5% [95% CI, 4.7%-6.4%] to 6.7% [95% CI, 5.7%-7.8%]; *P* = .09) and Tennessee (from 5.6% [95% CI, 4.6%-6.7%] to 6.9% [95% CI, 5.7%-8.5%]; *P* = .13) also recorded increases in the prevalence of e-cigarette use between 2018 and 2020, although these increases were not statistically significant (Table 2).

**Table 2.** Weighted Prevalence and Change in Absolute Prevalence of Current e-Cigarette Use by State Among US Adults in the Behavioral Risk Factor Surveillance System

State	Weighted prevalence, % (95% CI)			Difference in absolute prevalence, % (95% CI)		
	2017	2018	2020	2018 vs 2017	2020 vs 2018	2020 vs 2017
Alabama	4.9 (4.2 to 5.8)	NA	6.1 (5.1 to 7.2)	NA	NA	1.1 (−0.2 to 2.5)
Alaska	3.5 (2.4 to 5.0)	6.0 (4.7 to 7.7)	5.0 (3.9 to 6.4)	2.6 (0.6 to 4.5)	−1.1 (−3.0 to 0.9)	1.5 (−0.3 to 3.3)
Arizona	5.3 (4.8 to 5.8)	NA	NA	NA	NA	NA
Arkansas	5.7 (4.4 to 7.3)	7.0 (5.8 to 8.5)	5.7 (4.7 to 6.9)	1.4 (−0.6 to 3.3)	−1.3 (−3.1 to 0.4)	0.02 (−1.8 to 1.8)
California	3.0 (2.6 to 3.5)	NA	NA	NA	NA	NA
Colorado	5.3 (4.7 to 6.0)	7.5 (6.3 to 8.8)	NA	2.1 (0.7 to 3.5)	NA	NA
Connecticut	3.2 (2.7 to 3.8)	5.6 (4.9 to 6.4)	4.5 (3.8 to 5.2)	2.4 (1.4 to 3.3)	−1.1 (−2.1 to −0.1)	1.3 (0.4 to 2.1)
Delaware	4.8 (3.9 to 6.0)	4.7 (3.9 to 5.7)	4.6 (3.6 to 5.8)	−0.1 (−1.5 to 1.2)	−0.2 (−1.6 to 1.2)	−0.3 (−1.8 to 1.2)
District of Columbia	2.3 (1.7 to 3.1)	NA	NA	NA	NA	NA
Florida	4.3 (3.8 to 5.0)	5.9 (5.0 to 6.9)	5.7 (4.6 to 7.0)	1.6 (0.5 to 2.7)	−0.2 (−1.7 to 1.3)	1.3 (−0.01 to 2.7)
Georgia	4.4 (3.8 to 5.2)	5.3 (4.6 to 6.0)	5.6 (4.7 to 6.5)	0.8 (−0.2 to 1.8)	0.3 (−0.8 to 1.4)	1.1 (−0.02 to 2.3)
Guam	6.6 (5.1 to 8.5)	5.9 (4.5 to 7.9)	11.4 (8.7 to 14.8)	−0.7 (−3.0 to 1.7)	5.5 (2.0 to 9.0)	4.9 (1.4 to 8.3)
Hawaii	4.7 (4.0 to 5.4)	7.1 (6.1 to 8.1)	5.9 (5.1 to 6.8)	2.5 (1.3 to 3.6)	−1.2 (−2.4 to 0.02)	1.3 (0.2 to 2.4)
Idaho	4.6 (3.8 to 5.7)	5.8 (4.6 to 7.4)	6.7 (5.7 to 7.8)	1.2 (−0.5 to 2.9)	0.8 (−0.9 to 2.6)	2.0 (0.6 to 3.4)
Illinois	4.4 (3.6 to 5.3)	NA	3.4 (2.6 to 4.3)	NA	NA	−1.0 (−2.2 to 0.1)
Indiana	6.0 (5.4 to 6.7)	6.7 (5.8 to 7.7)	5.8 (5.1 to 6.5)	0.7 (−0.5 to 1.8)	−0.9 (−2.1 to 0.3)	−0.3 (−1.2 to 0.7)
Iowa	4.0 (3.5 to 4.7)	5.3 (4.7 to 6.0)	NA	1.3 (0.4 to 2.1)	NA	NA
Kansas	4.6 (4.2 to 5.0)	5.5 (4.7 to 6.4)	6.7 (5.7 to 7.8)	0.9 (−0.1 to 1.9)	1.2 (−0.2 to 2.5)	2.1 (0.9 to 3.2)
Kentucky	6.1 (5.1 to 7.2)	NA	6.7 (5.7 to 8.0)	NA	NA	0.7 (−0.9 to 2.2)
Louisiana	4.5 (3.7 to 5.4)	5.7 (4.8 to 6.7)	NA	1.2 (−0.1 to 2.5)	NA	NA
Maine	4.1 (3.4 to 4.9)	5.3 (4.1 to 6.8)	4.3 (3.5 to 5.2)	1.2 (−0.4 to 2.8)	−1.0 (−2.6 to 0.6)	0.2 (−0.9 to 1.3)
Maryland	3.3 (2.7 to 3.9)	4.3 (3.8 to 4.9)	3.8 (3.2 to 4.4)	1.0 (0.2 to 1.8)	−0.5 (−1.3 to 0.3)	0.5 (−0.3 to 1.3)
Massachusetts	3.3 (2.6 to 4.1)	5.6 (4.8 to 6.5)	4.1 (3.1 to 5.3)	2.3 (1.1 to 3.5)	−1.5 (−2.9 to −0.1)	0.8 (−0.5 to 2.1)
Michigan	4.9 (4.3 to 5.5)	6.1 (5.3 to 7.0)	6.4 (5.5 to 7.6)	1.2 (0.1 to 2.2)	0.4 (−1.0 to 1.7)	1.5 (0.3 to 2.8)
Minnesota	3.7 (3.3 to 4.1)	5.0 (4.6 to 5.5)	4.9 (4.4 to 5.4)	1.4 (0.7 to 2.0)	−0.2 (−0.9 to 0.6)	1.2 (0.5 to 1.9)
Mississippi	4.9 (4.0 to 6.1)	5.6 (4.8 to 6.6)	4.6 (3.9 to 5.5)	0.7 (−0.7 to 2.1)	−1.0 (−2.2 to 0.3)	−0.3 (−1.6 to 1.0)
Missouri	5.2 (4.4 to 6.0)	5.6 (4.6 to 6.7)	5.7 (5.0 to 6.5)	0.4 (−0.9 to 1.7)	0.2 (−1.1 to 1.4)	0.6 (−0.5 to 1.7)
Montana	3.9 (3.2 to 4.8)	4.7 (3.9 to 5.7)	4.9 (4.2 to 5.8)	0.8 (−0.4 to 2.0)	0.2 (−1.0 to 1.4)	1.0 (−0.1 to 2.2)
Nebraska	3.8 (3.3 to 4.4)	5.6 (5.0 to 6.3)	5.9 (5.2 to 6.6)	1.8 (0.9 to 2.7)	0.2 (−0.7 to 1.2)	2.0 (1.1 to 2.9)
Nevada	5.4 (4.3 to 6.8)	NA	6.7 (5.1 to 8.7)	NA	NA	1.2 (−0.9 to 3.4)
New Hampshire	4.6 (3.5 to 5.9)	4.7 (3.8 to 5.7)	5.5 (4.6 to 6.6)	0.1 (−1.4 to 1.6)	0.8 (−0.6 to 2.2)	0.9 (−0.6 to 2.5)
New Jersey	4.4 (3.8 to 5.3)	NA	5.0 (4.4 to 5.6)	NA	NA	0.5 (−0.5 to 1.5)
New Mexico	4.9 (4.0 to 5.9)	NA	5.6 (4.6 to 6.8)	NA	NA	0.7 (−0.8 to 2.2)
New York	3.8 (3.2 to 4.4)	5.4 (4.9 to 5.9)	4.1 (3.5 to 4.7)	1.6 (0.9 to 2.3)	−1.3 (−2.1 to −0.5)	0.3 (−0.5 to 1.1)
North Carolina	4.6 (3.7 to 5.6)	5.1 (4.2 to 6.2)	4.7 (4.0 to 5.5)	0.5 (−0.9 to 1.8)	−0.4 (−1.6 to 0.9)	0.1 (−1.1 to 1.3)
North Dakota	4.3 (3.5 to 5.2)	6.4 (5.3 to 7.6)	4.5 (3.6 to 5.6)	2.1 (0.6 to 3.5)	−1.9 (−3.4 to −0.3)	0.2 (−1.1 to 1.6)
Ohio	5.3 (4.7 to 6.1)	5.4 (4.7 to 6.1)	6.0 (5.4 to 6.7)	0.02 (−1.0 to 1.1)	0.7 (−0.4 to 1.7)	0.7 (−0.3 to 1.7)
Oklahoma	7.1 (6.2 to 8.1)	NA	NA	NA	NA	NA
Oregon	4.4 (3.7 to 5.2)	5.4 (4.7 to 6.2)	4.5 (3.9 to 5.3)	1.1 (−0.01 to 2.1)	−0.9 (−1.9 to 0.2)	0.2 (−0.9 to 1.2)
Pennsylvania	4.7 (4.0 to 5.5)	NA	5.3 (4.5 to 6.3)	NA	NA	0.6 (−0.5 to 1.8)
Puerto Rico	1.2 (0.8 to 1.7)	NA	NA	NA	NA	NA
Rhode Island	4.9 (4.0 to 6.0)	5.5 (4.6 to 6.7)	4.6 (3.7 to 5.8)	0.6 (−0.8 to 2.1)	−0.9 (−2.4 to 0.6)	−0.3 (−1.7 to 1.2)
South Carolina	4.1 (3.5 to 4.7)	NA	NA	NA	NA	NA
South Dakota	3.9 (2.9 to 5.4)	4.6 (3.5 to 6.1)	3.9 (3.0 to 5.3)	0.7 (−1.1 to 2.5)	−0.7 (−2.4 to 1.1)	0 (−1.7 to 1.7)
Tennessee	5.9 (4.9 to 6.9)	5.6 (4.6 to 6.7)	6.9 (5.7 to 8.5)	−0.3 (−1.7 to 1.2)	1.4 (−0.4 to 3.1)	1.1 (−0.6 to 2.8)
Texas	4.7 (3.9 to 5.7)	5.2 (4.3 to 6.3)	4.7 (3.9 to 5.6)	0.5 (−0.9 to 1.8)	−0.5 (−1.8 to 0.8)	−0.03 (−1.3 to 1.2)
Utah	5.1 (4.6 to 5.8)	6.1 (5.5 to 6.7)	7.2 (6.5 to 8.0)	0.9 (0.1 to 1.8)	1.2 (0.2 to 2.1)	2.1 (1.1 to 3.0)
Vermont	3.1 (2.3 to 4.0)	NA	4.0 (3.2 to 5.1)	NA	NA	1.0 (−0.3 to 2.3)
Virginia	4.9 (4.3 to 5.7)	4.9 (4.2 to 5.7)	5.2 (4.5 to 6.0)	−0.04 (−1.0 to 1.0)	0.3 (−0.8 to 1.3)	0.2 (−0.8 to 1.3)
Washington	4.3 (3.8 to 4.8)	NA	NA	NA	NA	1.0 (0.2 to 1.7)
West Virginia	5.7 (4.8 to 6.6)	NA	6.3 (5.4 to 7.4)	NA	NA	0.7 (−0.7 to 2.0)
Wisconsin	4.3 (3.5 to 5.2)	4.8 (4.0 to 5.9)	NA	0.6 (−0.7 to 1.8)	NA	NA
Wyoming	5.8 (4.8 to 6.9)	6.5 (5.4 to 7.7)	5.9 (4.8 to 7.3)	0.7 (−0.8 to 2.3)	−0.6 (−2.3 to 1.1)	0.2 (−1.4 to 1.8)

Abbreviation: NA, not available.

## Discussion

In this cross-sectional study using 2017, 2018, and 2020 BRFSS data, we observed that between 2017 and 2018, the prevalence of current e-cigarette use increased among US adults. However, between 2018 and 2020, a slight decrease was found in the prevalence of current e-cigarette use. This decrease was mainly observed among young adults aged 18 to 20 years. In addition, we found an increase in daily e-cigarette use, particularly among those aged 21 to 24 years, and an increase in the proportion of current users who use their device daily, which suggested that more e-cigarette users were becoming regular users. State-level patterns were heterogeneous but characterized mainly by significant decreases or nonsignificant changes in the prevalence of current e-cigarette use from 2018 to 2020 in most states, with the exception of states and territories like Utah and Guam.

Consistent with our findings, studies using data from the National Health Interview Survey^2,3^ also reported an increase in the prevalence of current e-cigarette use among US adults between 2017 and 2018. This increase, primarily observed in younger age groups, was associated with the concurrent rise in the availability of flavored products and high nicotine–concentration pod mod devices (modular vaping devices with refillable or replaceable nicotine cartridges, or pods, such as JUUL brand devices [JUUL Labs, Inc]).^20^ Findings from our study revealed that the prevalence of current e-cigarette use among US adults has subsequently decreased, which is similar to recent findings from the National Health Interview Survey.^21^ The modest reduction in e-cigarette use, particularly among adults younger than 21 years, may be an early sign of the consequences of several recently implemented federal and state policies. First, in December 2019, the federal minimum age for the sale of all tobacco products, including e-cigarettes, was raised from 18 years to 21 years (Tobacco-21 legislation), making it illegal for persons younger than 21 years to purchase tobacco products.^10,22^ In addition, the extensive public education and increased public awareness that accompanied reports of e-cigarette– or vaping product–associated lung injuries in 2019 may have been associated with this decrease. Miech et al^23^ reported that between 2019 and 2020, there was a decrease in the perceived accessibility of e-cigarettes and an increase in the perceived risk of vaping among US youths. The FDA ban on flavored cartridge–based e-cigarettes, which took effect in February 2020, may have been instrumental in the reduction in e-cigarette use among young adults because most youths and young adults report using flavored products.^8,24^ Moreover, the COVID-19 pandemic and associated stay-at-home orders may have been additional factors associated with the decrease in e-cigarette use.^25^

Although the observed decrease in current e-cigarette use between 2018 and 2020 may have represented an actual reduction, it is important to consider other factors that may have produced a spurious reduction. With the rapidly evolving e-cigarette language and the increasing market share of disposable e-cigarettes (such as the Puff Bar brand [Puff Bar]),^26^ it is unclear whether BRFSS survey questions fully captured these newer products. A recent study^27^ found that querying about general e-cigarette use (as in most surveys) rather than specific e-cigarette devices or brands, may underestimate the true prevalence of use.

Another notable observation was the increase in the prevalence of daily e-cigarette use, particularly among young adults aged 21 to 24 years. Although daily e-cigarette users previously represented a small proportion of e-cigarette users, this pattern has changed in recent years.^1,18^ We found a steady increase in the proportion of current e-cigarette users who reported daily use. This increase may imply that more e-cigarette users are becoming regular rather than experimental users. Taken together with the recent reduction in current e-cigarette use, this finding could mean that the decrease in current e-cigarette use is more reflective of quitting among experimental but not regular users. However, because of the cross-sectional nature of this study, we were unable to assess these transitions. The implications of frequent e-cigarette use may differ among combustible cigarette smokers and nonsmokers. Although more frequent e-cigarette use has been associated with nicotine dependence and a greater risk of combustible cigarette initiation, particularly among tobacco-naive adolescents,^28,29^ it has also been associated with greater odds of discontinuing combustible cigarette smoking and reductions in the number of cigarettes smoked per day among daily smokers.^30,31,32^

Among current combustible cigarette smokers who reported attempting to quit in the past year, the prevalence of current e-cigarette use decreased between 2018 and 2020, with only 13.1% reporting current e-cigarette use in 2020. Although the reasons for e-cigarette use were not explored in the present study, previous studies^33,34^ have reported that most older adult smokers of combustible cigarettes may use e-cigarettes to aid in tobacco cessation. The potential reasons for the decreasing prevalence of e-cigarette use among combustible cigarette smokers attempting to quit were unclear from our study data and need to be further explored; however, the reasons may pertain to decreasing belief in the effectiveness of e-cigarettes as cessation aids.

State-level patterns in current e-cigarette use were heterogeneous. The timing of the implementation of various e-cigarette policies varied from state to state and may have accounted for the observed differences.^35^ Between 2018 and 2020, significant reductions in current e-cigarette use were observed in Connecticut, Massachusetts, New York, and North Dakota. Our findings for Massachusetts and New York were consistent with recent decreases in e-cigarette sales in those states after statewide restrictions on flavored e-cigarette sales were implemented.^36^ In contrast, Guam and Utah recorded significant increases in current e-cigarette use between 2018 and 2020. Although Utah has the lowest prevalence of combustible cigarette smoking in the US,^37^ the prevalence of current e-cigarette use in that state has steadily increased. It is unclear whether e-cigarette use is replacing combustible cigarette use. Guam is among the states and territories that had already increased the minimum age for tobacco sales to 21 years before the implementation of the federal policy.^38^ However, the increasing prevalence of e-cigarette use in Guam may reflect the state’s less restrictive policies regarding e-cigarette excise taxes and packaging.^39^ States with increasing patterns of e-cigarette use might consider closely monitoring these patterns and strictly enforcing existing e-cigarette policies. Implementing other policies, such as a statewide flavor ban, and increasing e-cigarette taxes may help to reverse the observed patterns.^36^

Our findings have important regulatory implications. Although the prevalence of current e-cigarette use slightly decreased among young adults aged 18 to 20 years, a considerable proportion of this age group still reported using e-cigarettes. Therefore, continued surveillance and stricter enforcement of the Tobacco-21 legislation, the e-cigarette flavor ban, and marketing restrictions are warranted. Among young adults aged 21 to 24 years, regulations and policy interventions that make e-cigarette use less appealing to this age group, such as extending the flavor ban to all non–tobacco-flavored e-cigarettes and increasing the excise tax on these products, may be considered. In addition, the increase in daily e-cigarette use needs to be closely monitored and further investigated. Future studies assessing the sociodemographic and device characteristics associated with transitioning from occasional to daily or frequent e-cigarette use may guide targeted health promotion campaigns and inform regulatory policies.

### Limitations

This study has several limitations. First, all data were self-reported, with the potential for misclassification and recall bias. Second, in 2018 and 2020, not all states provided data on e-cigarette use, limiting the generalizability of our findings. However, we observed similar patterns in the prevalence of e-cigarette use when restricting analyses to only states with e-cigarette data for all 3 years, adding to the robustness of our findings. Third, because of the cross-sectional nature of our study, we could not assess transitions (eg, whether occasional e-cigarette users were transitioning to frequent use). Fourth, the observational nature of this study did not allow us to determine the factors contributing to the observed changes in e-cigarette use. Although we have provided some potential reasons for the modest reduction in e-cigarette use between 2018 and 2020, such as the Tobacco-21 legislation, continued surveillance over the next several years will be important to assess the consequences of these policy changes.

## Conclusions

This cross-sectional study found that the prevalence of current (past 30 days) e-cigarette use slightly decreased among US adults between 2018 and 2020, mainly among young adults aged 18 to 20 years. However, daily e-cigarette use, particularly among young adults aged 21 to 24 years, increased, warranting continued surveillance. Notably, the proportion of e-cigarette users who reported daily e-cigarette use increased significantly between 2017 and 2020, which was likely reflective of overall patterns of greater nicotine dependence among those who used e-cigarettes.

## Notes

Supplement. **eMethods.** Assessment of e-Cigarette and Combustible Cigarette Use and Other Study Measures **eTable 1.** States Reporting Data on e-Cigarette Use in 2017, 2018, and 2020, Behavioral Risk Factor Surveillance System **eTable 2.** Patterns in Current and Daily e-Cigarette Use, Behavioral Risk Factor Surveillance System—Sensitivity Analyses Using Only the 33 States With e-Cigarette Data in 2017, 2018, and 2020 **eTable 3.** Patterns in Current e-Cigarette Use Prevalence Among Never Combustible Cigarette Smokers by Age, Behavioral Risk Factor Surveillance System (2017, 2018, and 2020) **eFigure 1.** Weighted Prevalence of Current Combustible Cigarette Use Among States With Data on e-Cigarette Use by Age, Behavioral Risk Factor Surveillance System (2017, 2018, and 2020) **eFigure 2.** Weighted Prevalence of the Patterns of e-Cigarette and Combustible Cigarette Use, Behavioral Risk Factor Surveillance System (2020) **eFigure 3.** Patterns in the Prevalence of Dual e-Cigarette and Combustible Cigarette Use by Age, Behavioral Risk Factor Surveillance System (2017, 2018, and 2020) **eFigure 4.** Prevalence of Current e-Cigarette Use by State, Behavioral Risk Factor Surveillance System (2017) **eFigure 5.** Prevalence of Current e-Cigarette Use by State, Behavioral Risk Factor Surveillance System (2018) **eFigure 6.** Prevalence of Current e-Cigarette Use by State, Behavioral Risk Factor Surveillance System (2020)

## References

[1] Obisesan OH, Osei AD, Uddin SMI, . Trends in e-cigarette use in adults in the United States, 2016-2018. JAMA Intern Med. 2020;180(10):1394-1398. doi:10.1001/jamainternmed.2020.2817 32897288 PMC7489391

[2] Dai H, Leventhal AM. Prevalence of e-cigarette use among adults in the United States, 2014-2018. *JAMA*. 2019;322(18):1824-1827. doi:10.1001/jama.2019.15331 PMC6749536 31524940

[3] Bao W, Liu B, Du Y, Snetselaar LG, Wallace RB. Electronic cigarette use among young, middle-aged, and older adults in the United States in 2017 and 2018. JAMA Intern Med. 2020;180(2):313-314. doi:10.1001/jamainternmed.2019.4957 31609399 PMC6802265

[4] US Department of Health and Human Services. Patterns of e-cigarette use among U.S. youth and young adults. In: *E-Cigarette Use Among Youth and Young Adults: a Report of the Surgeon General*. US Department of Health and Human Services, Centers for Disease Control and Prevention, National Center for Chronic Disease Prevention and Health Promotion, and Office on Smoking and Health; 2016: 27-93. Accessed July 19, 2020. https://www.ncbi.nlm.nih.gov/books/NBK538680/pdf/Bookshelf_NBK538680.pdf

[5] Arrazola RA, Singh T, Corey CG, ; Centers for Disease Control and Prevention (CDC). Tobacco use among middle and high school students—United States, 2011–2014. *MMWR Morb Mortal Wkly Rep*. 2015;64(14):381-385. PMC5779546 25879896

[6] Gentzke AS, Creamer M, Cullen KA, . Vital signs: tobacco product use among middle and high school students—United States, 2011-2018. MMWR Morb Mortal Wkly Rep. 2019;68(6):157-164. doi:10.15585/mmwr.mm6806e1 30763302 PMC6375658

[7] Park-Lee E, Ren C, Sawdey MD, . Notes from the field: e-cigarette use among middle and high school students—National Youth Tobacco Survey, United States, 2021. MMWR Morb Mortal Wkly Rep. 2021;70(39):1387-1389. doi:10.15585/mmwr.mm7039a4 34591834 PMC8486384

[8] Wang TW, Gentzke AS, Neff LJ, . Characteristics of e-cigarette use behaviors among US youth, 2020. JAMA Netw Open. 2021;4(6):e2111336. doi:10.1001/jamanetworkopen.2021.11336 34097049 PMC8185598

[9] Tobacco to 21 Act, HR 2411, 116th Cong (2019-2020). Accessed June 12, 2022. https://www.congress.gov/bill/116th-congress/house-bill/2411/text

[10] Newly signed legislation raises federal minimum age of sale of tobacco products to 21. News release. US Food and Drug Administration. January 15, 2020. Accessed October 22, 2021. https://www.fda.gov/tobacco-products/ctp-newsroom/newly-signed-legislation-raises-federal-minimum-age-sale-tobacco-products-21

[11] FDA finalizes enforcement policy on unauthorized flavored cartridge-based e-cigarettes that appeal to children, including fruit and mint. News release. US Food and Drug Administration. January 2, 2020. Accessed July 17, 2020. https://www.fda.gov/news-events/press-announcements/fda-finalizes-enforcement-policy-unauthorized-flavored-cartridge-based-e-cigarettes-appeal-children

[12] Office on Smoking and Health, National Center for Chronic Disease Prevention and Health Promotion. Outbreak of lung injury associated with the use of e-cigarette, or vaping, products. Centers for Disease Control and Prevention; 2020. Updated February 25, 2020. Accessed July 12, 2020. https://www.cdc.gov/tobacco/basic_information/e-cigarettes/severe-lung-disease.html

[13] FDA permits marketing of e-cigarette products, marking first authorization of its kind by the agency. News release. US Food and Drug Administration. October 12, 2021. Accessed October 22, 2021. https://www.fda.gov/news-events/press-announcements/fda-permits-marketing-e-cigarette-products-marking-first-authorization-its-kind-agency

[14] Centers for Disease Control and Prevention. Weighting the BRFSS data. Centers for Disease Control and Prevention; 2017. Accessed July 3, 2020. https://www.cdc.gov/brfss/annual_data/2017/pdf/weighting-2017-508.pdf

[15] Centers for Disease Control and Prevention. The Behavioral Risk Factor Surveillance System: 2017 summary data quality report. Centers for Disease Control and Prevention. June 13, 2018. Accessed December 17, 2020. https://www.cdc.gov/brfss/annual_data/2017/pdf/2017-sdqr-508.pdf

[16] Centers for Disease Control and Prevention. Behavioral Risk Factor Surveillance System: 2018 summary data quality report. Centers for Disease Control and Prevention. July 17, 2019. Accessed December 17, 2020. https://www.cdc.gov/brfss/annual_data/2018/pdf/2018-sdqr-508.pdf

[17] Centers for Disease Control and Prevention. Behavioral Risk Factor Surveillance System: 2020 summary data quality report. Centers for Disease Control and Prevention. August 2, 2021. Accessed December 17, 2020. https://www.cdc.gov/brfss/annual_data/2020/pdf/2020-sdqr-508.pdf

[18] Mirbolouk M, Charkhchi P, Kianoush S, . Prevalence and distribution of e-cigarette use among U.S. adults: Behavioral Risk Factor Surveillance System, 2016. *Ann Intern Med*. 2018;169(7):429-438. doi:10.7326/M17-3440 PMC10534294 30167658

[19] Li C, Ford ES, Zhao G, Wen XJ, Gotway CA. Age adjustment of diabetes prevalence: use of 2010 U.S. census data. J Diabetes. 2014;6(5):451-461. doi:10.1111/1753-0407.12122 24393518 PMC11287713

[20] Romberg AR, Miller Lo EJ, Cuccia AF, . Patterns of nicotine concentrations in electronic cigarettes sold in the United States, 2013-2018. Drug Alcohol Depend. 2019;203:1-7. doi:10.1016/j.drugalcdep.2019.05.029 31386973 PMC6765364

[21] Cornelius ME, Loretan CG, Wang TW, Jamal A, Homa DM. Tobacco product use among adults—United States, 2020. MMWR Morb Mortal Wkly Rep. 2022;71(11):397-405. doi:10.15585/mmwr.mm7111a1 35298455 PMC8942309

[22] Center for Tobacco Products. Tobacco 21: update for Retailers. US Food and Drug Administration; 2019. Accessed October 25, 2021. https://www.fda.gov/media/151919/download

[23] Miech R, Leventhal A, Johnston L, O’Malley PM, Patrick ME, Barrington-Trimis J. Trends in use and perceptions of nicotine vaping among US youth from 2017 to 2020. JAMA Pediatr. 2021;175(2):185-190. doi:10.1001/jamapediatrics.2020.5667 33320241 PMC7739194

[24] Office on Smoking and Health, National Center for Chronic Disease Prevention and Health Promotion. *E-Cigarette Use Among Youth and Young Adults: a Report of the Surgeon General*. Centers for Disease Control and Prevention; 2016. Accessed June 28, 2020. https://www.ncbi.nlm.nih.gov/books/NBK538684/

[25] Gaiha SM, Lempert LK, Halpern-Felsher B. Underage youth and young adult e-cigarette use and access before and during the coronavirus disease 2019 pandemic. JAMA Netw Open. 2020;3(12):e2027572. doi:10.1001/jamanetworkopen.2020.27572 33270127 PMC7716191

[26] CDC Foundation. Monitoring U.S. e-cigarette sales: national trends. Data brief. CDC Foundation; July 2021. Accessed November 11, 2021. https://www.cdcfoundation.org/National-E-CigaretteSales-DataBrief-2021-July11?inline

[27] Morean ME, Camenga DR, Bold KW, . Querying about the use of specific e-cigarette devices may enhance accurate measurement of e-cigarette prevalence rates among high school students. Nicotine Tob Res. 2020;22(5):833-837. doi:10.1093/ntr/nty240 30395344 PMC7171282

[28] Morean ME, Krishnan-Sarin S, S O’Malley S. Assessing nicotine dependence in adolescent e-cigarette users: the 4-item Patient-Reported Outcomes Measurement Information System (PROMIS) Nicotine Dependence Item Bank for electronic cigarettes. Drug Alcohol Depend. 2018;188:60-63. doi:10.1016/j.drugalcdep.2018.03.029 29753155 PMC6983293

[29] Harlow AF, Stokes AC, Brooks DR, Benjamin EJ, Barrington-Trimis JL, Ross CS. E-cigarette use and combustible cigarette smoking initiation among youth: accounting for time-varying exposure and time-dependent confounding. Epidemiology. 2022;33(4):523-532. doi:10.1097/EDE.0000000000001491 35394965 PMC9156560

[30] Kasza KA, Edwards KC, Kimmel HL, . Association of e-cigarette use with discontinuation of cigarette smoking among adult smokers who were initially never planning to quit. JAMA Netw Open. 2021;4(12):e2140880. doi:10.1001/jamanetworkopen.2021.40880 34962556 PMC8715340

[31] Pearson JL, Zhou Y, Smiley SL, . Intensive longitudinal study of the relationship between cigalike e-cigarette use and cigarette smoking among adult cigarette smokers without immediate plans to quit smoking. Nicotine Tob Res. 2021;23(3):527-534. doi:10.1093/ntr/ntaa086 32421191 PMC7885790

[32] Berry KM, Reynolds LM, Collins JM, . E-cigarette initiation and associated changes in smoking cessation and reduction: the Population Assessment of Tobacco and Health Study, 2013-2015. Tob Control. 2019;28(1):42-49. 29574448 10.1136/tobaccocontrol-2017-054108 PMC6317439

[33] Patel D, Davis KC, Cox S, . Reasons for current e-cigarette use among U.S. adults. Prev Med. 2016;93:14-20. doi:10.1016/j.ypmed.2016.09.011 27612572 PMC5316292

[34] Romijnders KAGJ, van Osch L, de Vries H, Talhout R. Perceptions and reasons regarding e-cigarette use among users and non-users: a narrative literature review. Int J Environ Res Public Health. 2018;15(6):1190. doi:10.3390/ijerph15061190 29882828 PMC6025300

[35] Preventing Tobacco Addiction Foundation. Tobacco 21: the law of the land. Preventing Tobacco Addiction Foundation; 2021. Accessed December 20, 2021. https://tobacco21.org/

[36] Ali FRM, Vallone D, Seaman EL, . Evaluation of statewide restrictions on flavored e-cigarette sales in the US from 2014 to 2020. JAMA Netw Open. 2022;5(2):e2147813. doi:10.1001/jamanetworkopen.2021.47813 35142832 PMC8832173

[37] Report reveals Utah has lowest smoking rate in U.S. News release. Utah Now. September 8, 2021. Accessed May 2, 2022. https://utahnow.online/2021/09/08/report-reveals-utah-has-lowest-smoking-rate-in-u-s/

[38] Marynak K, Mahoney M, Williams KAS, Tynan MA, Reimels E, King BA. State and territorial laws prohibiting sales of tobacco products to persons aged <21 years—United States, December 20, 2019. MMWR Morb Mortal Wkly Rep. 2020;69(7):189-192. doi:10.15585/mmwr.mm6907a3 32078593 PMC7043390

[39] Public Health Law Center. E-cigarette regulations—Guam. Public Health Law Center at Mitchell Hamline School of Law; 2022. Accessed May 2, 2022. https://www.publichealthlawcenter.org/resources/us-e-cigarette-regulations-50-state-review/gu

