# Supplementary material for: Assessment of Patterns in e-Cigarette Use Among Adults in the US, 2017-2020
Source: JAMA Netw Open. 2022 Jul 22;5(7):e2223266. doi: 10.1001/jamanetworkopen.2022.23266 (PMC9308055; doi:10.1001/jamanetworkopen.2022.23266)
Supplement: Supplement. — eMethods. Assessment of e-Cigarette and Combustible Cigarette Use and Other Study Measures eTable 1. States Reporting Data on e-Cigarette Use in 2017, 2018, and 2020, Behavioral Risk Factor Surveillance System eTable 2. Patterns in Current and Daily e-Cigarette Use, Behavioral Risk Factor Surveillance System—Sensitivity Analyses Using Only the 33 States With e-Cigarette Data in 2017, 2018, and 2020 eTable 3. Patterns in Current e-Cigarette Use Prevalence Among Never Combustible Cigarette Smokers by Age, Behavioral Risk Factor Surveillance System (2017, 2018, and 2020) eFigure 1. Weighted Prevalence of Current Combustible Cigarette Use Among States With Data on e-Cigarette Use by Age, Behavioral Risk Factor Surveillance System (2017, 2018, and 2020) eFigure 2. Weighted Prevalence of the Patterns of e-Cigarette and Combustible Cigarette Use, Behavioral Risk Factor Surveillance System (2020) eFigure 3. Patterns in the Prevalence of Dual e-Cigarette and Combustible Cigarette Use by Age, Behavioral Risk Factor Surveillance System (2017, 2018, and 2020) eFigure 4. Prevalence of Current e-Cigarette Use by State, Behavioral Risk Factor Surveillance System (2017) eFigure 5. Prevalence of Current e-Cigarette Use by State, Behavioral Risk Factor Surveillance System (2018) eFigure 6. Prevalence of Current e-Cigarette Use by State, Behavioral Risk Factor Surveillance System (2020) [file jamanetwopen-e2223266-s001.pdf]

Supplementary Online Content

Boakye E, Osuji N, Erhabor J, et al. Assessment of patterns in e-cigarette use among adults in the US, 2017-2020. *JAMA Netw Open*. 2022;5(7):e2223266. doi:10.1001/jamanetworkopen.2022.23266

eMethods. Assessment of e-Cigarette and Combustible Cigarette Use and Other Study Measures

eTable 1. States Reporting Data on e-Cigarette Use in 2017, 2018, and 2020, Behavioral Risk Factor Surveillance System

eTable 2. Patterns in Current and Daily e-Cigarette Use, Behavioral Risk Factor Surveillance System—Sensitivity Analyses Using Only the 33 States With e-Cigarette Data in 2017, 2018, and 2020

eTable 3. Patterns in Current e-Cigarette Use Prevalence Among Never Combustible Cigarette Smokers by Age, Behavioral Risk Factor Surveillance System (2017, 2018, and 2020)

eFigure 1. Weighted Prevalence of Current Combustible Cigarette Use Among States With Data on e-Cigarette Use by Age, Behavioral Risk Factor Surveillance System (2017, 2018, and 2020)

eFigure 2. Weighted Prevalence of the Patterns of e-Cigarette and Combustible Cigarette Use, Behavioral Risk Factor Surveillance System (2020)

eFigure 3. Patterns in the Prevalence of Dual e-Cigarette and Combustible Cigarette Use by Age, Behavioral Risk Factor Surveillance System (2017, 2018, and 2020)

eFigure 4. Prevalence of Current e-Cigarette Use by State, Behavioral Risk Factor Surveillance System (2017)

eFigure 5. Prevalence of Current e-Cigarette Use by State, Behavioral Risk Factor Surveillance System (2018)

eFigure 6. Prevalence of Current e-Cigarette Use by State, Behavioral Risk Factor Surveillance System (2020)

This supplementary material has been provided by the authors to give readers additional information about their work.

© 2022 Boakye E et al. *JAMA Network Open*.

eMethods. Assessment of e-Cigarette and Combustible Cigarette Use and Other Study Measures

Assessment of e-Cigarette and Combustible Cigarette Use

E-cigarette use was assessed with the questions, “Have you ever used an e-cigarette or other electronic vaping product, even just one time, in your entire life?” and “Do you now use e-cigarettes or other electronic vaping products every day, some days, or not at all?”. Participants who responded “yes” to the first question and answered “every day” or “some days” to the second question were considered current e-cigarette users. Current e-cigarette users were further categorized by their frequency of use into daily and occasional users.

Similarly, combustible cigarette use was categorized as never, former, and current based on answers to the questions “Have you smoked at least 100 cigarettes in your entire life? and “Do you now smoke cigarettes every day, some days, or not at all?” Among current combustible cigarette smokers, past-12-month quit attempt was assessed with the question, “During the past 12 months, have you stopped smoking for one day or longer because you were trying to quit smoking?”

Other Study Measures

Sociodemographic characteristics included age, sex (male, female), race/ethnicity (American Indian/Alaskan Native, Hispanic, Native Hawaiian/Pacific Islander, non-Hispanic Asian, non-Hispanic Black, non-Hispanic White, Multi-racial, other), sexual orientation (heterosexual, lesbian/gay, bisexual), transgender identity (yes, no), marital status (married, divorced, widowed, single, member of an unmarried couple), highest education level completed (<high school, high school/some college, college graduate), employment (employed, unemployed, student, retired), and income level. Race/ethnicity categories were obtained from participant responses to the questions, “Are you Hispanic, Latino/a, or Spanish origin?” and “Which one or more of the following would you say is your race?”. Options to the second question included White, Black or African American, American Indian or Alaskan Native, Asian, Pacific Islander, and other. Annual family income was adjusted using federal poverty guidelines and categorized as: below 100%, within 100-200%, or above 200% of the poverty line.[17] Body mass index (BMI) was calculated from self-reported weight and height (<18.5, 18.5-<25.0, 25.0-<30.0, and ≥30.0kg/m^2).

Use of other substances, including smokeless tobacco products (yes, no), past-30-day cannabis use (yes, no), heavy alcohol use (yes, no), and binge drinking (yes, no), were also included. Heavy alcohol use was defined as consuming >14 drinks per week for men or >7 drinks per week for women, and binge drinking as consuming on a single occasion ≥5 drinks for men or ≥4 drinks for women. Chronic health conditions assessed included cardiovascular diseases (CVD; composite of myocardial infarction, coronary heart disease, or stroke), asthma, chronic obstructive pulmonary disease (COPD), depression, and cancer.

© 2022 Boakye E et al. *JAMA Network Open*.

eTable 1. States Reporting Data on e-Cigarette Use in 2017, 2018, and 2020, Behavioral Risk Factor Surveillance System

Year	Number of states	States with data on e-cigarettes	Count	Data on current e-cigarette use
2017	ALL	All states, District of Columbia, Guam, Puerto Rico	450,016	429,370
2018	37	Alaska, Arkansas, Connecticut, Delaware, Florida, Georgia, Guam, Hawaii, Idaho, Indiana, Iowa, Louisiana, Maryland, Massachusetts, Minnesota, Mississippi, Missouri, Montana, Nebraska, New Hampshire, New York, North Carolina, North Dakota, Ohio, Oregon, Rhode Island, South Dakota, Tennessee, Texas, Utah, Virginia, Wisconsin, Wyoming, Colorado, Kansas, Maine, Michigan	307,985	280,184
2020	43	Alabama, Alaska, Arkansas, Connecticut, Delaware, Florida, Georgia, Guam, Hawaii, Idaho, Illinois, Indiana, Kentucky, Maine, Maryland, Minnesota, Mississippi, Missouri, Montana, Nebraska, Nevada, New Hampshire, New Jersey, New Mexico, North Carolina, North Dakota, Ohio, Oregon, Pennsylvania, Rhode Island, South Dakota, Tennessee, Texas, Utah, Vermont, Virginia, Washington, West Virginia, Wyoming, Kansas, Massachusetts, Michigan, New York	322,426	284,753

© 2022 Boakye E et al. *JAMA Network Open*.

eTable 2. Patterns in Current and Daily e-Cigarette Use, Behavioral Risk Factor Surveillance System—Sensitivity Analyses Using Only the 33 States With e-Cigarette Data in 2017, 2018, and 2020

	Weighted prevalence, % (95% CI)			Absolute prevalence differences ((95% CI)		
	2017 (Unweighted n=282,639)	2018 (Unweighted n=259,543)	2020 (Unweighted n=228,659)	2018 vs 2017	2020 vs 2018	2020 vs 2017
Current Use						
Overall	4.5 (4.4-4.7)	5.5 (5.3-5.7)	5.2 (4.9-5.4)	1.0 (0.7 - 1.3)	-0.4 (-0.7 - -0.1)	0.6 (0.3 - 0.9)
Age groups, years						
18-20	11.9 (10.6-13.4)	18.6 (16.8-20.5)	15.5 (13.8-17.3)	6.7 (4.4 - 9.0)	-3.1 (-5.7 - -0.6)	3.5 (1.3 - 5.8)
21-24	9.4 (8.3-10.7)	13.3 (12.1-14.6)	14.5 (13.0-16.1)	3.9 (2.1 - 5.6)	1.2 (-0.8 - 3.2)	5.1 (3.1 - 7.1)
25-29	6.7 (6.1-7.5)	9.2 (8.3-10.2)	9.8 (8.6-11.2)	2.5 (1.3 - 3.6)	0.6 (-1.0 - 2.2)	3.1 (1.6 - 4.6)
30-34	6.0 (5.4-6.8)	8.2 (7.3-9.2)	8.0 (7.1-9.1)	2.2 (1.0 - 3.3)	-0.2 (-1.5 - 1.2)	2.0 (0.8 - 3.2)
35-39	5.2 (4.6-5.9)	7.6 (6.7-8.6)	6.2 (5.4-7.1)	2.4 (1.3 - 3.6)	-1.4 (-2.7 - -0.1)	1.0 (-0.1 - 2.1)
40-44	4.6 (3.8-5.4)	4.6 (4.0-5.2)	4.4 (3.8-5.1)	0.0 (-1.0 - 1.1)	-0.2 (-1.1 - 0.7)	-0.1 (-1.2 - 0.9)
45-49	3.6 (3.1-4.2)	4.7 (4.0-5.4)	3.3 (2.6-4.1)	1.1 (0.2 - 1.9)	-1.4 (-2.4 - -0.4)	-0.4 (-1.2 - 0.6)
50-54	3.8 (3.3-4.4)	3.5 (3.0-4.1)	3.3 (2.8-3.8)	-0.3 (-1.1 - 0.4)	-0.3 (-1.0 - 0.5)	-0.6 (-1.3 - 0.2)
55-59	3.3 (2.8-3.9)	2.9 (2.6-3.3)	2.8 (2.4-3.3)	-0.4 (-1.0 - 0.2)	-0.1 (-0.7 - 0.5)	-0.5 (-1.2 - 0.2)
>=60	1.6 (1.4-1.7)	1.5 (1.4-1.7)	1.3 (1.1-1.5)	-0.1 (-0.3 - 0.2)	-0.2 (-0.5 - 0.0)	-0.3 (-0.5 - -0.1)
Daily Use						
Overall	1.5 (1.4-1.6)	2.1 (1.9-2.2)	2.3 (2.2-2.5)	0.5 (0.3 - 0.7)	0.3 (0.1 - 0.5)	0.8 (0.6 - 1.0)
Age groups, years						
18-20	3.5 (2.8-4.3)	7.1 (6.0-8.5)	6.0 (5.0-7.2)	3.6 (2.2 - 5.1)	-1.1 (-2.8 - 0.6)	2.5 (1.2 - 3.9)
21-24	2.7 (2.2-3.4)	4.4 (3.7-5.1)	6.9 (5.8-8.2)	1.7 (0.7 - 2.6)	2.5 (1.2 - 3.9)	4.2 (2.9 - 5.5)
25-29	2.4 (2.0-2.9)	3.1 (2.6-3.6)	4.0 (3.3-4.9)	0.6 (0.0 - 1.3)	1.0 (0.0 - 1.9)	1.6 (0.7 - 2.5)
30-34	1.8 (1.5-2.1)	3.2 (2.6-3.9)	3.8 (3.2-4.5)	1.4 (0.7 - 2.2)	0.6 (-0.4 - 1.5)	2.0 (1.3 - 2.8)
35-39	1.9 (1.6-2.2)	3.4 (2.8-4.1)	3.2 (2.5-3.9)	1.5 (0.8 - 2.2)	-0.2 (-1.2 - 0.7)	1.3 (0.5 - 2.1)
40-44	1.7 (1.3-2.1)	1.8 (1.5-2.2)	2.1 (1.7-2.6)	0.2 (-0.4 - 0.8)	0.3 (-0.3 - 0.9)	0.5 (-0.2 - 1.1)
45-49	1.4 (1.1-1.8)	1.6 (1.2-2.2)	1.7 (1.2-2.4)	0.2 (-0.4 - 0.8)	0.0 (-0.7 - 0.8)	0.2 (-0.5 - 0.9)
50-54	1.4 (1.1-1.7)	1.2 (1.0-1.5)	1.5 (1.2-1.9)	-0.2 (-0.6 - 0.3)	0.3 (-0.2 - 0.8)	0.2 (-0.3 - 0.6)
55-59	1.2 (0.9-1.6)	1.1 (0.9-1.3)	1.2 (0.9-1.6)	-0.1 (-0.5 - 0.3)	0.1 (-0.3 - 0.5)	0.0 (-0.5 - 0.5)
>=60	0.6 (0.5-0.8)	0.6 (0.5-0.7)	0.6 (0.5-0.7)	-0.1 (-0.2 - 0.1)	0.0 (-0.2 - 0.2)	0.0 (-0.2 - 0.13)

© 2022 Boakye E et al. *JAMA Network Open*.

eTable 3. Patterns in Current e-Cigarette Use Prevalence Among Never Combustible Cigarette Smokers by Age, Behavioral Risk Factor Surveillance System (2017, 2018, and 2020)

Age groups, years	Weighted prevalence, % (95% CI)			Absolute prevalence differences (95% CI)		
	2017 (Unweighted n= 243,677)	2018 (Unweighted n= 158,272)	2020 (Unweighted n= 165,220)	2018 vs. 2017	2020 vs. 2018	2020 vs. 2017
18-20	8.4 (7.4-9.4)	14.4 (12.8-16.1)	12.0 (10.7-13.5)	6.0 (4.1 - 7.9)	-2.4 (-4.5 - -0.2)	3.6 (1.9 - 5.4)
21-24	3.8 (3.2-4.5)	7.4 (6.4-8.5)	8.7 (7.6-9.9)	3.5 (2.3 - 4.8)	1.3 (-0.2 - 2.9)	4.9 (3.6 - 6.2)
25-29	2.5 (2.0-3.0)	3.2 (2.6-3.8)	4.0 (3.1-5.1)	0.7 (0.0 - 1.5)	0.8 (-0.4 - 1.9)	1.5 (0.4 - 2.6)
30-34	1.2 (0.9-1.5)	1.8 (1.5-2.3)	2.8 (2.1-3.7)	0.7 (0.2 - 1.2)	1.0 (0.1 - 1.8)	1.6 (0.8 - 2.4)
35-39	0.8 (0.6-1.2)	1.3 (0.9-1.9)	1.4 (1.0-1.9)	0.5 (0.0 - 1.1)	0.0 (-0.6 - 0.7)	0.6 (0.1 - 1.1)
40-44	0.4 (0.3-0.7)	0.7 (0.5-1.0)	0.8 (0.5-1.3)	0.3 (0.0 - 0.6)	0.1 (-0.4 - 0.6)	0.4 (-0.1 - 0.8)
45-49	0.4 (0.2-0.6)	0.5 (0.3-0.8)	0.5 (0.3-0.8)	0.1 (-0.2 - 0.5)	0.0 (-0.4 - 0.3)	0.1 (-0.2 - 0.4)
50-54	0.2 (0.1-0.3)	0.5 (0.3-0.8)	0.5 (0.3-0.7)	0.3 (0.1 - 0.5)	0.0 (-0.3 - 0.2)	0.3 (0.1 - 0.5)
55-59	0.2 (0.1-0.3)	0.3 (0.2-0.5)	0.4 (0.3 - 0.6)	0.1 (0.0 - 0.3)	0.1 (-0.1 - 0.3)	0.2 (0.0 - 0.4)
>=60	0.1 (0.0-0.1)	0.2 (0.1-0.2)	0.2 (0.1-0.2)	0.1 (0.0 - 0.2)	0.0 (-0.1 - 0.1)	0.1 (0.0 - 0.2)

© 2022 Boakye E et al. *JAMA Network Open*.

eFigure 1. Weighted Prevalence of Current Combustible Cigarette Use Among States With Data on e-Cigarette Use by Age, Behavioral Risk Factor Surveillance System (2017, 2018, and 2020)

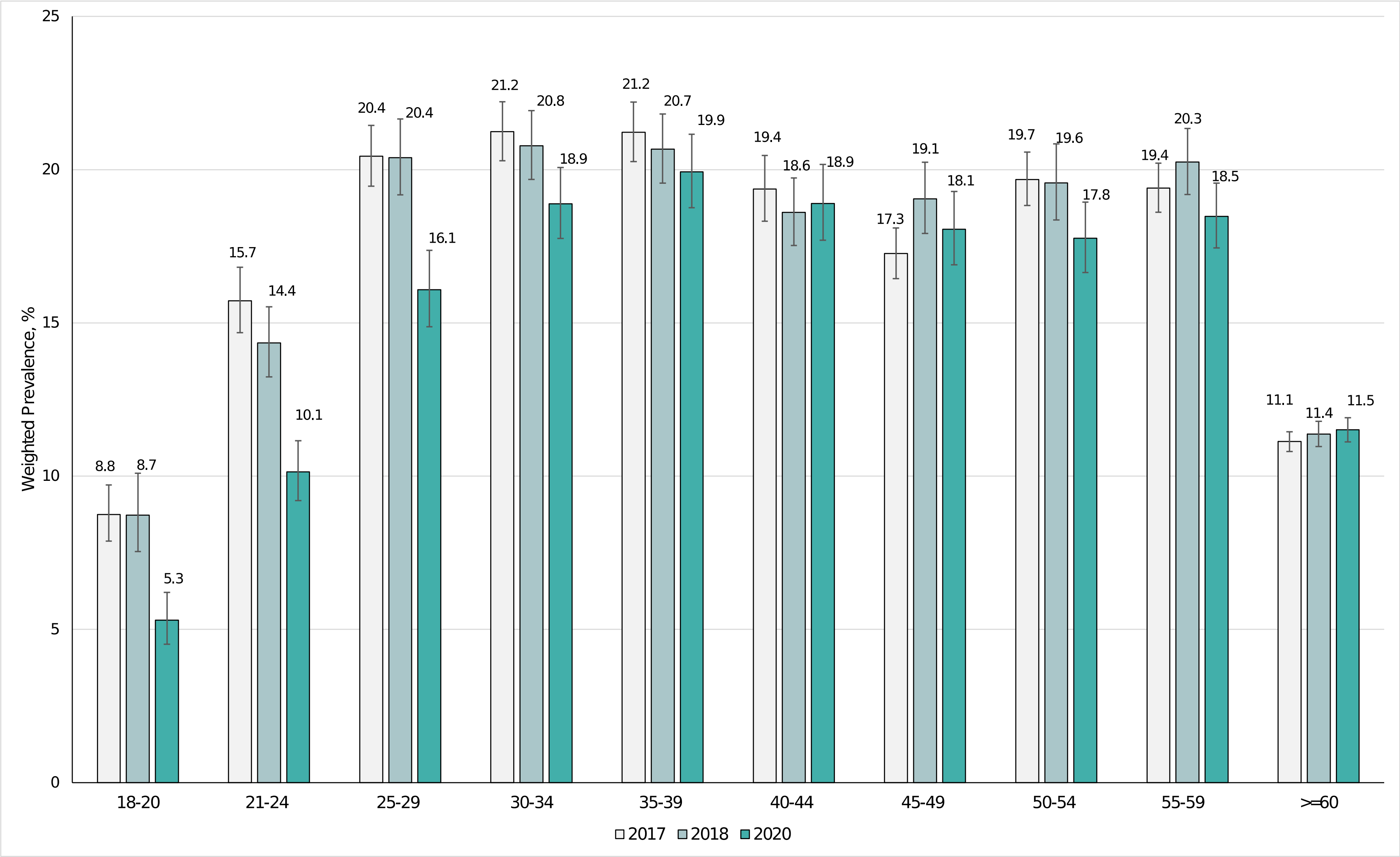

© 2022 Boakye E et al. *JAMA Network Open*.

eFigure 2. Weighted Prevalence of the Patterns of e-Cigarette and Combustible Cigarette Use, Behavioral Risk Factor Surveillance System (2020)

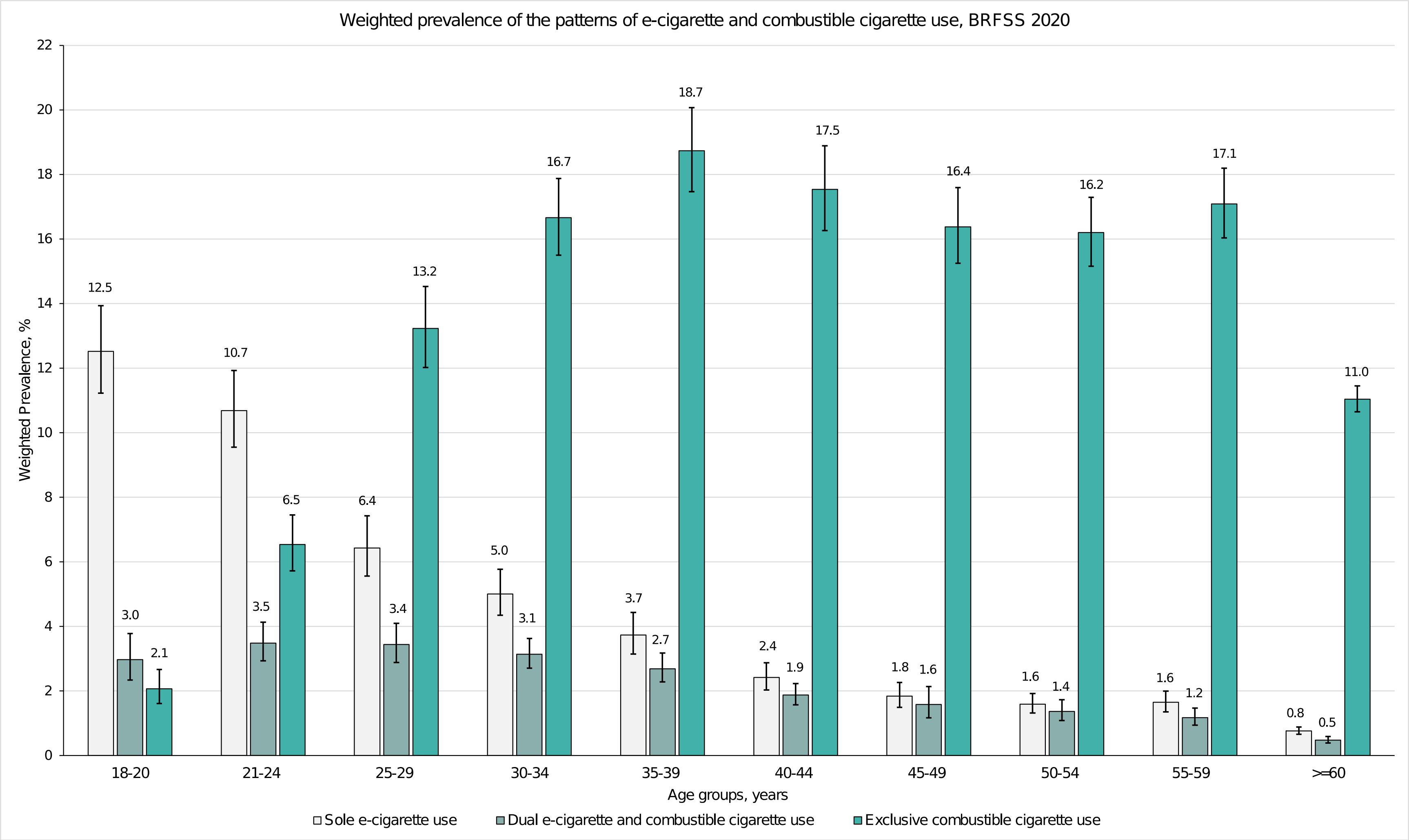

© 2022 Boakye E et al. *JAMA Network Open*.

eFigure 3. Patterns in the Prevalence of Dual e-Cigarette and Combustible Cigarette Use by Age, Behavioral Risk Factor Surveillance System (2017, 2018, and 2020)

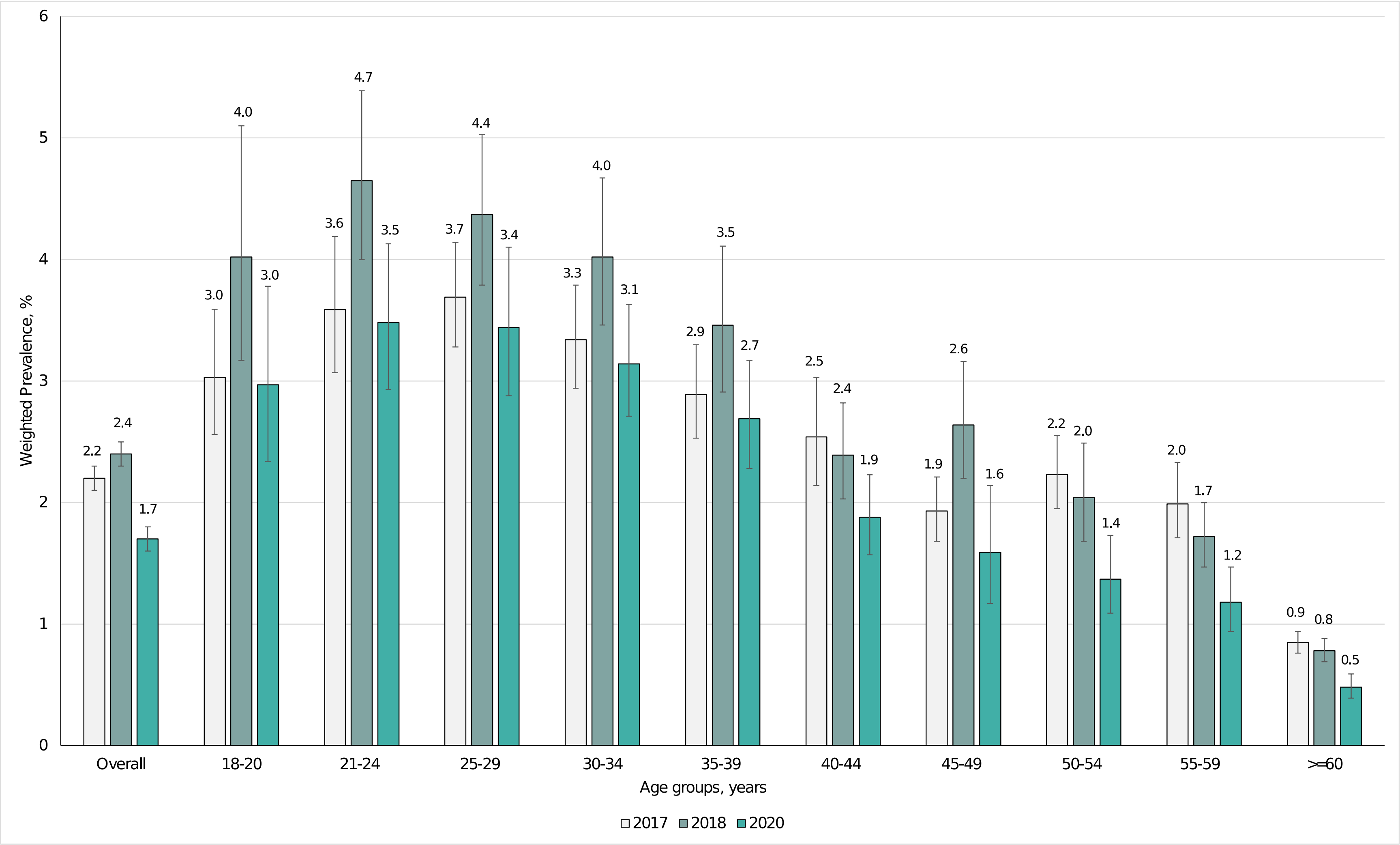

© 2022 Boakye E et al. *JAMA Network Open*.

eFigure 4. Prevalence of Current e-Cigarette Use by State, Behavioral Risk Factor Surveillance System (2017)

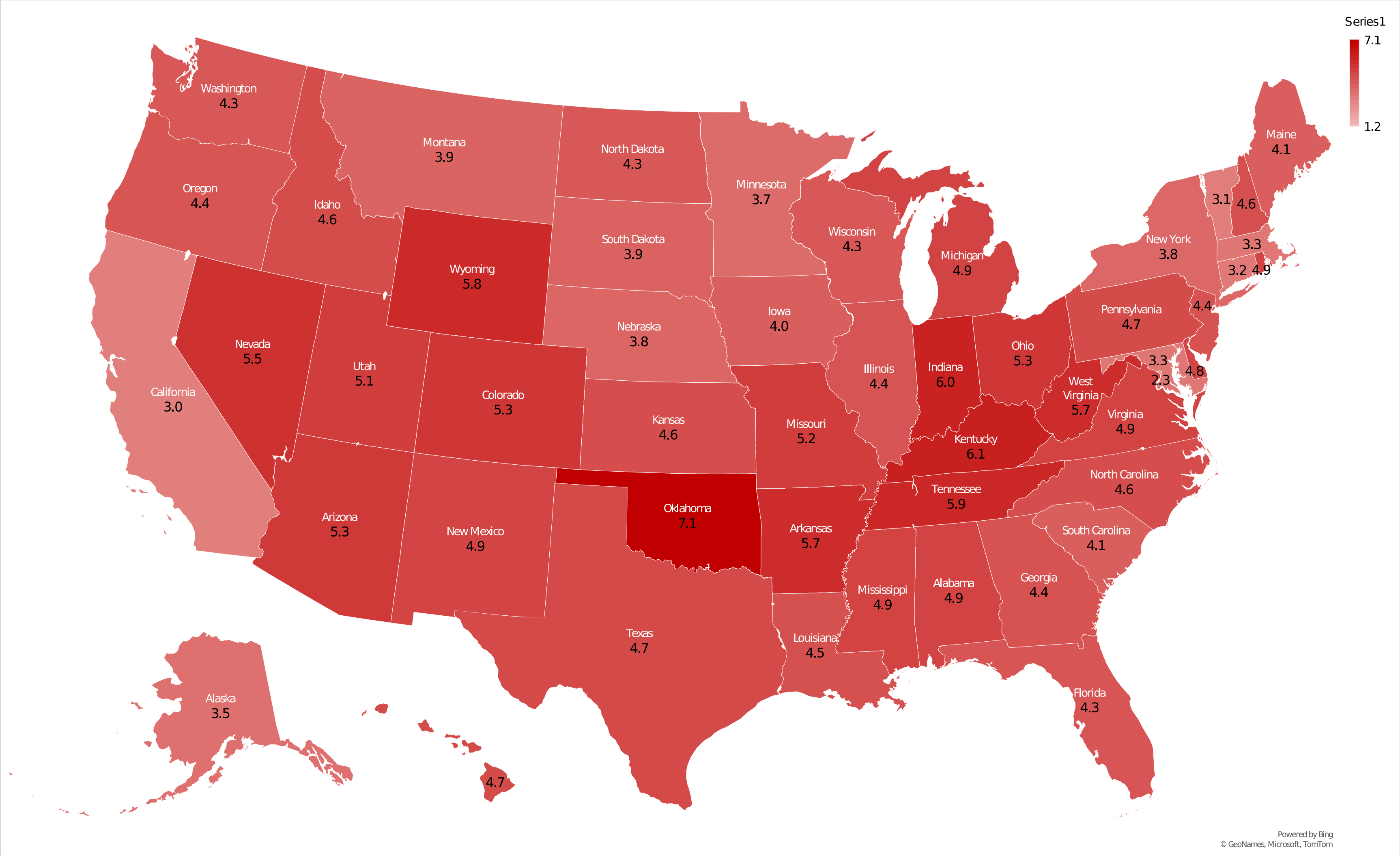

© 2022 Boakye E et al. *JAMA Network Open*.

eFigure 5. Prevalence of Current e-Cigarette Use by State, Behavioral Risk Factor Surveillance System (2018)

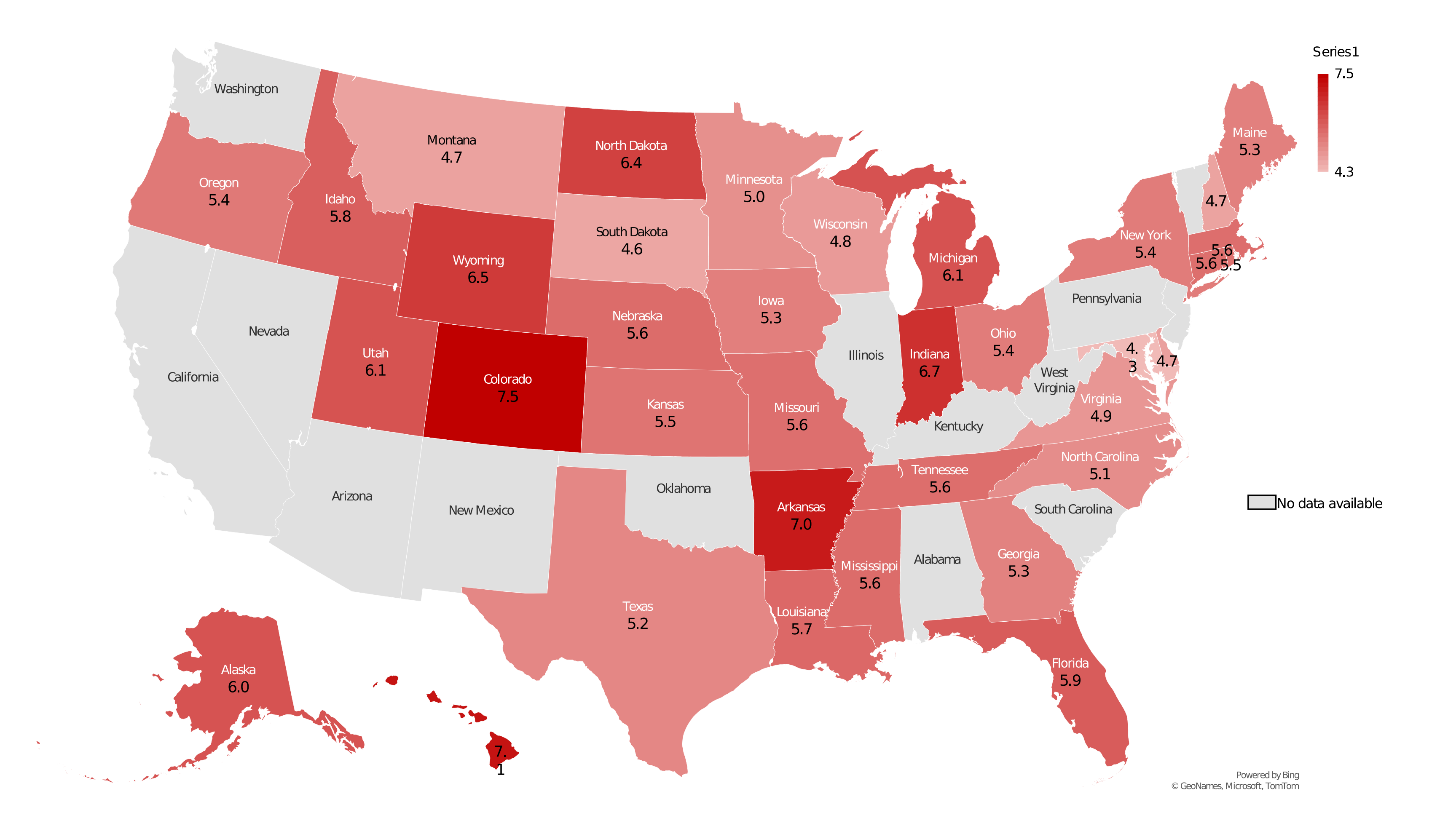

© 2022 Boakye E et al. *JAMA Network Open*.

eFigure 6. Prevalence of Current e-Cigarette Use by State, Behavioral Risk Factor Surveillance System (2020)

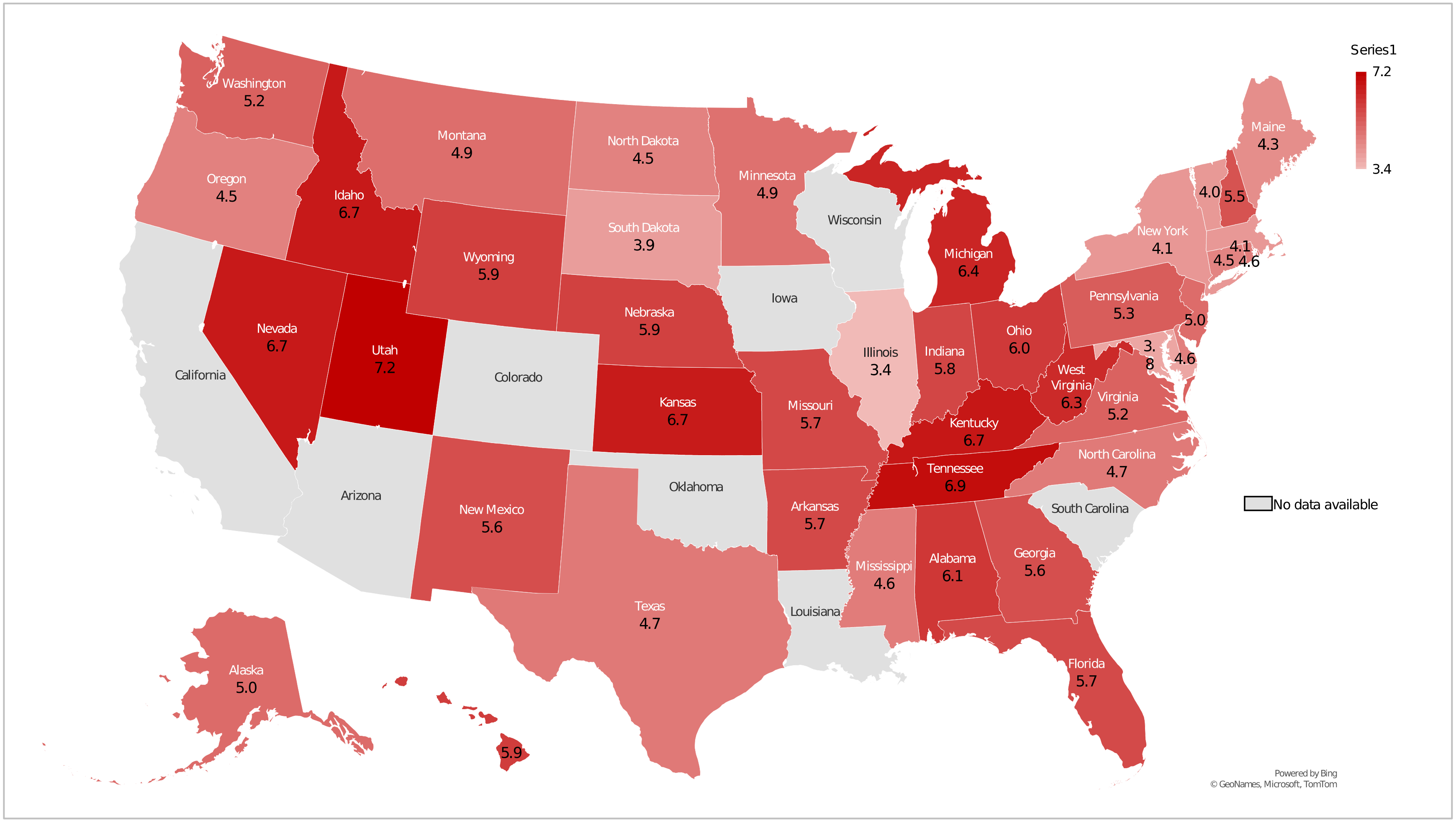

© 2022 Boakye E et al. *JAMA Network Open*.
